# Antibiotic Prescribing Patterns in Paediatric Primary Care in Italy: Findings from 2012–2018

**DOI:** 10.3390/antibiotics11010018

**Published:** 2021-12-24

**Authors:** Elisa Barbieri, Costanza di Chiara, Paola Costenaro, Anna Cantarutti, Carlo Giaquinto, Yingfen Hsia, Daniele Doná

**Affiliations:** 1 Division of Paediatric Infectious Diseases, Department for Woman and Child Health, University of Padua, 35100 Padua, Italy; Costanza.dichiara@phd.unipd.it (C.d.C.); paola.costenaro@phd.unipd.it (P.C.); carlo.giaquinto@unipd.it (C.G.); daniele.dona@unipd.it (D.D.); 2 Unit of Biostatistics, Epidemiology and Public Health, Department of Statistics and Quantitative Methods, University of Milano-Bicocca, 20126 Milan, Italy; anna.cantarutti@unimib.it; 3 National Centre for Healthcare Research and Pharmacoepidemiology, Department of Statistics and Quantitative Methods, University of Milano-Bicocca, 20126 Milan, Italy; 4 Società Servizi Telematici–Pedianet, 35121 Padua, Italy; 5 School of Pharmacy, Queen’s University Belfast, Belfast BT9 7BL, UK; Y.Hsia@qub.ac.uk; 6 Paediatric Infectious Disease Research Group, St. George’s University of London, London SW17 0RE, UK

**Keywords:** antibiotic prescriptions, children, primary care, treatment switch, Italy

## Abstract

Comprehensive data are needed to monitor antibiotic prescribing and inform stewardship. We aimed to evaluate the current antibiotic prescribing patterns, including treatment switching and prolongation, in the paediatric primary care setting in Italy. This database study assessed antibiotic prescriptions retrieved from Pedianet, a paediatric primary care database, from 1 January 2012 to 31 December 2018. Descriptive analyses were stratified by diagnosis class, calendar year, and children’s age. Generalized linear Poisson regression was used to assess variation in the prescriptions. In total, 505,927 antibiotic prescriptions were included. From 2012 to 2018, the number of antibiotics per child decreased significantly by 4% yearly from 0.79 in 2012 to 0.62 in 2018. Amoxicillin prescriptions decreased with increasing children’s age, while macrolides and third-generation cephalosporins had the opposite trend. Prescriptions were associated with a diagnosis of upper respiratory infection in 23% of cases, followed by pharyngitis (21%), bronchitis and bronchiolitis (12%), and acute otitis media (12%). Eight percent of treatment episodes were prolonged or switched class, mostly represented by co-amoxiclav, macrolides, and third-generation cephalosporins. Our findings report an overall decrease in antibiotic prescriptions, but pre-schoolers are still receiving more than one antibiotic yearly, and broad-spectrum antibiotics prescription rates remain the highest.

## 1. Introduction

Italy is one of the countries with the highest rate of antibiotic prescribing for children in Europe [1,2,3], as well as one with the highest antibiotic resistance rates [4]. In Italian paediatric primary care setting, antibiotics represent nearly 50% of overall medicines reimbursed by the National Healthcare System (NHS), of which penicillin combined with beta-lactamase inhibitors represent 38.4% of total reimbursed medicines, followed by cephalosporins (22.4%) and macrolides (18.8%) [5].

Studies have demonstrated that around 20 to 50% of antibiotic prescriptions are unnecessary or inappropriate [6,7,8], with patients have received broad-spectrum antibiotics for viral infections [9,10] or antibiotic treatment courses significantly longer than needed [11].

This unnecessary exposure to antibiotics may subsequently increase the risk of serious drug side effects and costs [12]. It has also strongly contributed to the emerging antibiotic resistance. It has been recommended that a reduction in antibiotic prescribing in adults and children could decrease the transmission of resistant strains [13,14].

In order to understand the scope and the burden of antibiotic prescriptions in Italy, comprehensive data are needed. Despite several studies having been carried out in hospitals [3,4,5,6,7,8,9,10,11,12,13,14,15] and community settings in certain regions [16,17,18,19,20,21], it is not possible to make a direct comparison due to different indicators (e.g., the percentage of children receiving amoxicillin as the first prescription or the percentage of children receiving macrolides or cephalosporins as first prescription [15]) and index unit analysed (e.g., first antibiotic prescription in a determined year [18] or oral formulations dispensed [21]). Moreover, comprehensive data on the patients and on the diagnoses associated with prescriptions have never been reported in the Italian paediatric primary care setting due to limitations in the administrative databases for capturing the medical information related to the medications.

Information captured in population datasets is valuable, as it will enable us to monitor antibiotic prescribing patterns. It will also assist in implementing antibiotic stewardship and informing policymakers.

The primary aim of our study was to comprehensively explore the current antibiotic prescription patterns in the paediatric primary care setting in Italy over the years, by child’s age and by diagnosis. Secondly, we described the treatment switch and prolongation patterns between different antibiotic classes.

## 2. Results

**Figure 1:**
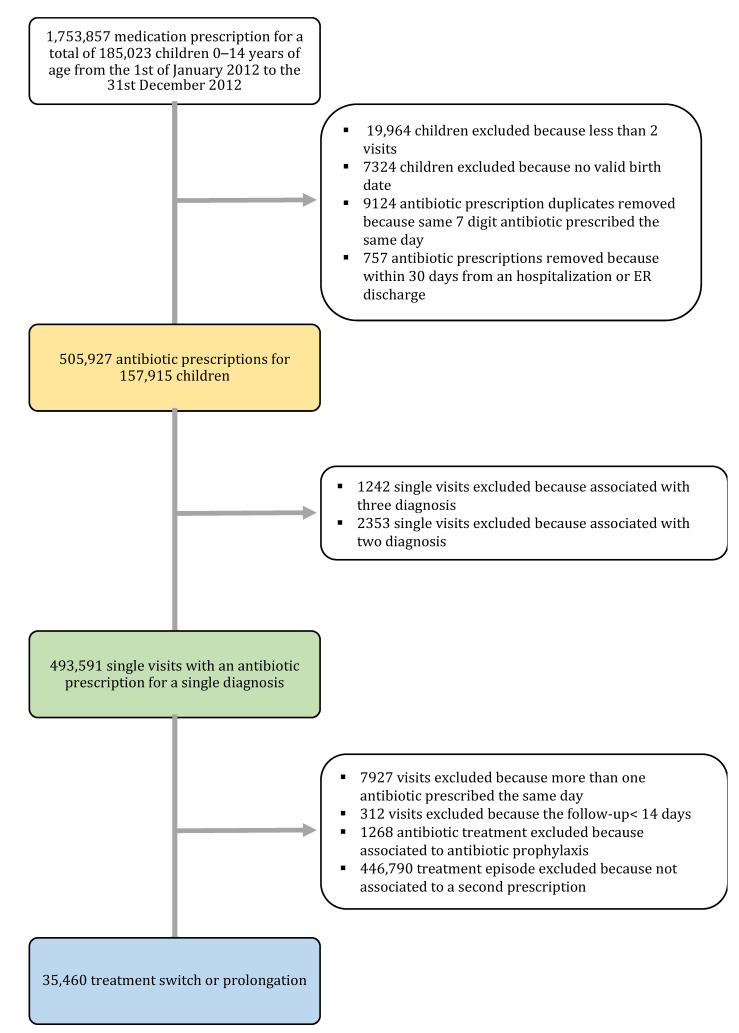
Flow chart of the data included and excluded from the different analyses. The yellow box represents the overall data used for the antibiotic index and prescription rate analysis stratified by age class and calendar year. The green box represents the overall data used for the prescription rate analysis stratified by age class, calendar year, and a single diagnosis. The light blue box represents the overall treatment episodes considered for the evaluation of treatment switching and prolongation.

In total, 157,915 children (51.9% males), for a total of 717,202 person-years of follow-up, were included. Overall, the median age was 7 years and most of the children resided in the north of Italy (Piemonte, Lombardy, Friuli Ven.-Giulia, and Veneto regions). The median number of children managed by each FP was 1144 (IQR: 640). In total, 505,927, prescriptions were considered. Descriptive data are presented in Table S1 in the Supplementary Material. The flow chart with the inclusion and exclusion criteria for the different outcomes is presented in Figure 1.

### 2.1. Antibiotic Prescribing Trend over Years and by Age Class

**Figure 2:**
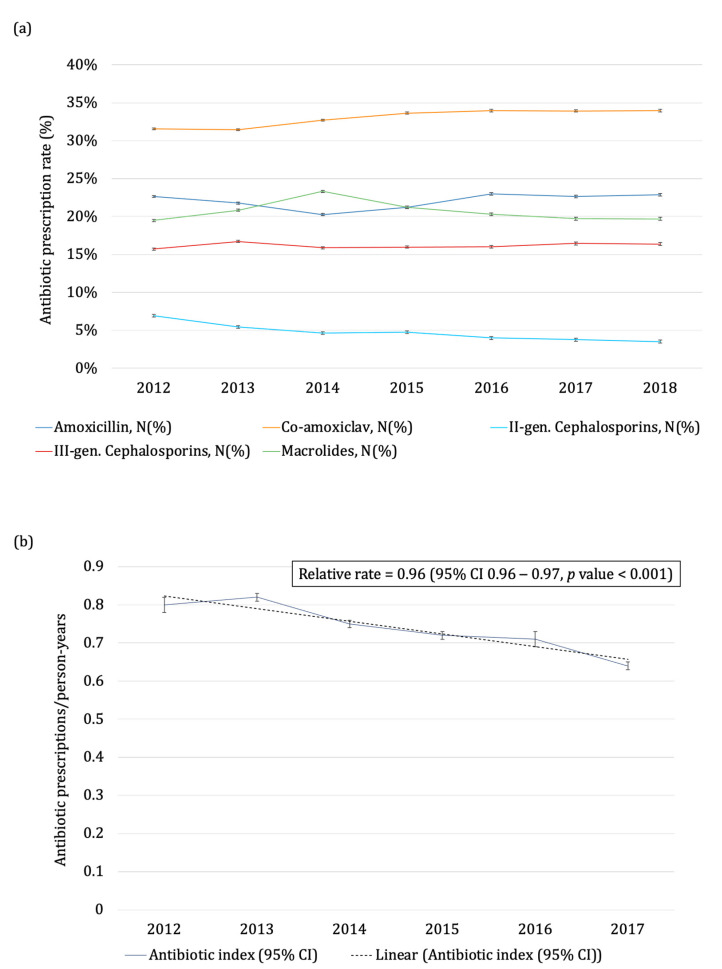
Antibiotic prescription rates of the different antibiotic classes (Panel (**a**)) and antibiotic indices (Panel (**b**)) by calendar year (Pedianet 2012–2018). Footnotes: The whiskers represent the relative 95% confidence intervals, and the dotted black line in Panel (**b**) represents the linear trend of the antibiotic index. Only antibiotic classes with a prescription rate value greater than 5% are reported.

From 2012 to 2018, the AI rate decreased significantly (*p* = 0.004) from 0.80 prescriptions per child in a year (95% CI: 0.78–0.81) in 2012 to 0.64 (95% CI: 0.63–0.65) in 2018, with an annual 4% reduction (relative rate: 0.96; 95% CI 0.96–0.97) (Figure 2 and Supplementary Material Table S1).

The highest AIs were reported for children aged from 1 to 4 years, with values around 1.2; on average, for every year of increase in the patient’s age, the AI was reduced by 13% (relative rate 0.87; 95% CI, 0.87–0.87, Supplementary Material Figure S1. Male children received 5% more antibiotics with respect to females, and patients residing in the centre or south of Italy received around 70% more antibiotics with respect to children residing in the northern regions (Tables S1 and S2).

When years of age were considered as a category in the model, estimates reported that children aged less than 7 years were at a higher risk of receiving an antibiotic compared with children less than 1 year of age. Children over 7 years of age were at a lower risk of receiving an antibiotic compared with infants (Supplementary Material Figure S1 and Table S2).

Co-amoxiclav, amoxicillin, macrolides, and third-generation cephalosporins accounted for 90% of all prescriptions (Figure 2 and Supplementary Material Table S1).

In the overall population, co-amoxiclav and amoxicillin prescription rates increased by 1% and 2% yearly, from 31.5% to 34% and from 22.7% to 22.9%, respectively (Table 1).

**Figure 3:**
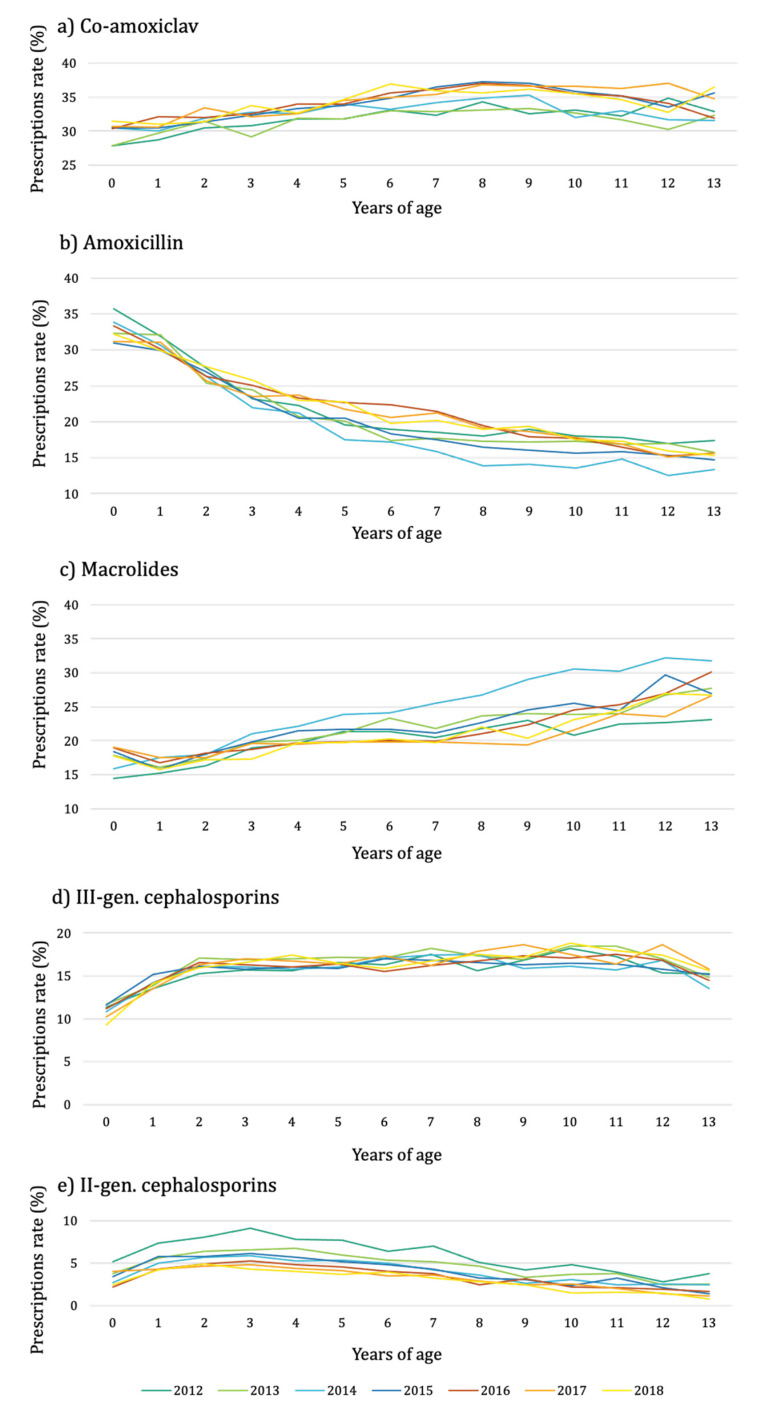
Antibiotic prescription rate stratified by years of age, antibiotic class (co-amoxiclav, Panel (**a**); amoxicillin, Panel (**b**); macrolides, Panel (**c**); third-generation cephalosporins, Panel (**d**); second-generation cephalosporins, Panel (**e**)), and calendar year, 2012–2018. Only antibiotic classes with a prescription rate value greater than 5% are reported.

The amoxicillin and second-generation cephalosporin prescription rates decreased with an increase in years of age (by −6% and −7%, respectively), from a maximum of 36% in 2012 for children <1 year of age to a minimum of 14% in 2014 for children aged 13 years for amoxicillin and from a maximum of 9% in 2012 for children aged 3 years to a minimum of 1% in 2018 for children aged 13 years for second-generation cephalosporins (Figure 3 and Supplementary Material Table S3).

An increasing trend with increasing age was noticed for co-amoxiclav, third-generation cephalosporins, and macrolides (by 1%, 1%, and 4%, respectively). Co-amoxiclav rates varied from a minimum of 28% in 2012 for children age <1 year to a maximum of 37% in 2017 for children aged 12 years; for second-generation cephalosporins, the rates varied from a minimum of 9% in 2018 in infants to a maximum of 18% in 2017 in 12-year-old children. In 2014, the macrolide rates were the highest with respect to the different calendar years in children aged 3–13 years (Figure 3).

Males were at a higher risk of receiving co-amoxiclav and macrolides (3% and 4%, respectively), and children residing in the centre and the south of Italy were at a higher risk of receiving co-amoxiclav and cephalosporins compared with children in the northern regions, who were at a higher risk of receiving amoxicillin (Table 1).

### 2.2. Antibiotic Prescribing Trend by Diagnosis Class

Of a total of 497,186 visits associated with an antibiotic prescription, 3595 visits were excluded because they had more than one diagnosis associated with the prescription.

**Figure 4:**
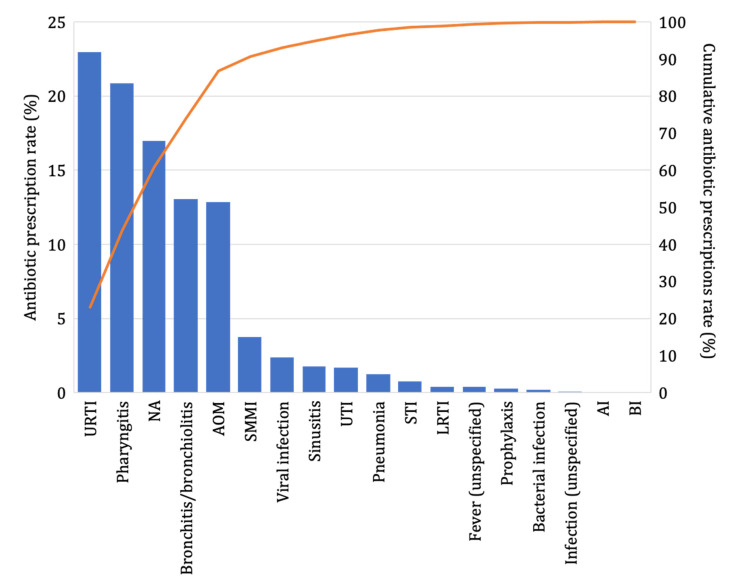
Antibiotic prescriptions’ prevalence rate for all prescriptions, stratified by diagnosis class with the cumulative rate slope shown in orange (Pedianet, 2012–2018). AOM = acute otitis media; URTI = upper respiratory tract infection; LRTI = lower respiratory tract infection; UTI = urinary tract infection; SMMI = skin and mucus membrane infection; STI = soft tissue infection; BI = bone infection; AI = abdominal infection.

Respiratory tract infections accounted for around 75% of the antibiotic prescriptions. Antibiotics were associated with a diagnosis of upper respiratory tract infection (URTI) in 23% of cases (*n* = 113,518), followed by diagnoses of pharyngitis (21%, *n* = 103,145), bronchitis and bronchiolitis (12%, *n* = 64,500), and acute otitis media (AOM, 12%, *n* = 63,527). In 84,006 prescriptions (16%), no diagnosis was associated with the antibiotic (Figure 4).

**Figure 5:**
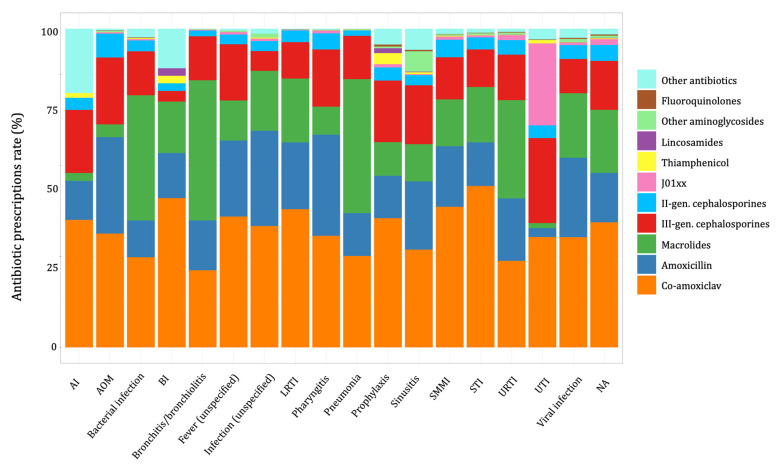
Antibiotic prescriptions’ prevalence rates, stratified by diagnosis class (Pedianet, 2012–2018). AOM = acute otitis media; URTI = upper respiratory tract infection; LRTI = lower respiratory tract infection; UTI = urinary tract infection; SMMI = skin and mucus membrane infection; STI = soft tissue infection; BI = bone infection; AI = abdominal infection.

Co-amoxiclav prescription rates varied from 51% for soft tissue infections (STI) to 24% for bronchitis and bronchiolitis. The amoxicillin prescription rate was 32% for pharyngitis, 30% for AOM, and 20% for URTI. Twenty-seven percent of prescriptions for urinary tract infections (UTIs) were for third-generation cephalosporins and J01XX class antibiotics (e.g., fosfomycin) (Figure 5 and Supplementary Material Table S4).

The prescription rates for the different diagnosis classes stratified by calendar year and years of age are presented in Figure S2 (Supplementary Material). Similar to the overall antibiotic trend, for respiratory tract infections, a decrease in amoxicillin prescriptions, with a consequent increase in macrolide prescriptions, is notable.

### 2.3. Antibiotic Treatment Switching and Prolongation

Overall, 485,976 had just one diagnosis associated with one antibiotic prescription and 312 were excluded because the follow-up was shorter than 14 days. The 485,664 visits were grouped by 448,058 treatment episodes, and 1268 were excluded because they were associated with antibiotic prophylaxis.

In total, 35,460 treatment switches or prolongations were included. Treatment episodes with at least one switch were 17,044, of which 453 had two or more switches. Treatment episodes with at least one prolonged antibiotic numbered 19,516. No difference in the diagnosis prevalence compared with overall prescriptions was noted (Supplementary Material Figure S3).

**Figure 6:**
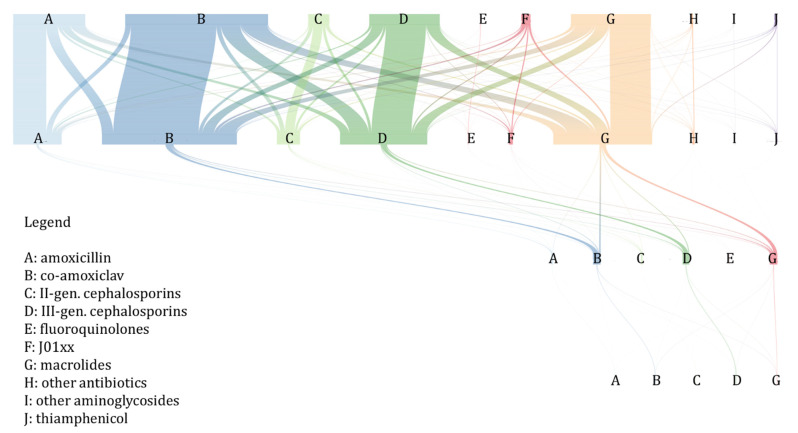
River plot of antibiotic treatment switching and prolongation. The rows of letters represent the prescriptions grouped by antibiotic classes in timely order from the upper part (first prescription) to the bottom part (last prescription), and the coloured lines show the connection with the following prescriptions in the same treatment episode (Pedianet, 2012–2018).

Overall, the antibiotic that was switched the most was co-amoxiclav (*n* = 5750 times, 31% of overall switches), especially for macrolides (*n* = 2402 times, 14%) and third-generation cephalosporins (*n* = 1792, 10%). The second most frequent class of antibiotics that was switched was macrolides (*n* = 4002, 21%), followed by amoxicillin (*n* = 3690, 20%). (Figure 6 and Supplementary Material Table S5).

The antibiotics that were prolonged the most were co-amoxiclav (N = 8132, 22% of the overall treatment changes), followed by third-generation cephalosporins (N = 3890) and macrolides (N = 3900), which each accounted for 10% of the overall treatment switches or prolongation (Supplementary material Table S5).

When stratified by diagnosis, the most frequent switch was for the combination of macrolides and co-amoxiclav for URTI, which accounted for 15% of the total switches for URTI.

Overall, 3358 antibiotics were switched before the fourth day (early switch), while the rest were classified as late switches. Co-amoxiclav was switched with a third-generation cephalosporin in 123 cases of pharyngitis, which was the most common early switch overall. The antibiotic class that was switched the most after the fourth day was the macrolides, which were substituted with co-amoxiclav in 577 episodes of URTI (Table 2).

Overall, the mean day of treatment switch was the eighth (SD: 4.0), with variations based on the antibiotic class accounting for the switch and the diagnosis (Table 2). In total, 4056 were considered to be early switches, and no variation was noted in the choice of the second antibiotic class. Interestingly, the switch from co-amoxiclav to third-generation cephalosporins for UTI was preferred over the choice of other antibiotic classes early in the treatment (Table 2).

## 3. Discussion

To our knowledge, this is the first longitudinal study to comprehensively assess antibiotic prescribing patterns in paediatric primary care in Italy. Over the period 2012–2018, we observed a decrease in total antibiotic prescriptions. Male children and those residing in central or southern regions were at a higher risk of receiving an antibiotic. In the overall population, co-amoxiclav and amoxicillin prescription rates increased over the years, with co-amoxiclav increasing, and also with an increase in the child’s age, and amoxicillin decreasing with an increase in the child’s age. Children residing in northern Italy were at a higher risk of receiving amoxicillin than those in the central and southern regions. Co-amoxiclav was the most prescribed antibiotic for respiratory infections (bronchitis/bronchiolitis, pharyngitis, URTI), accounting for half of the prescriptions. The prescribing patterns remained similar, with minimal variations between years of age over time. Overall, 8% of antibiotic treatment episodes were prolonged or switched in class, especially among co-amoxiclav, macrolides, and third-generation cephalosporins.

Assessing the drivers for the overprescribing of broad-spectrum antibiotics in the community is crucial, especially nowadays, since antibiotic resistance is dangerously rising globally. Indeed, many ecological studies showed a population-level association between antibiotic use and resistance [22]. Goossens et al. reported a significant variation across different countries in antibiotics use and bacterial resistance rates, with the highest rates in southern Europe [23]. Another study showed that after implementing new policies regarding outpatient antibiotic therapy, with a 42% decrease in macrolide use, there was a 48% reduction in the prevalence of macrolide-resistant Group A streptococci [24].

In line with previous studies [1,5], Italian children are receiving antibiotics at a rate 3.5 to 6 times higher than children in northern Europe. However, when we compare data from different countries, numerous factors should be considered. First, there is evidence suggesting that not all prescribed antibiotics are dispensed, and this may vary from country to country, depending on different determinants, including the socio-economic status of the family and the healthcare insurance that might affect the prescribing behaviour [25]. Second, different policy interventions targeting antibiotic use were implemented in various countries in Europe in different years [26]. As a result, the antibiotic prescribing patterns were impacted at a different level by these policy interventions. For example, in countries such as Denmark, where, in 2015, less than one antibiotic per child was prescribed yearly [27], a reimbursement policy targeting patients has been in place since the late 1990s, consisting of a reduced reimbursement rate for antibiotics purchased [28]; in Slovenia, the country with the highest rate of narrow-spectrum antibiotic consumption within total antibiotic consumption in 2015 [29], a policy is in place restricting the prescription of co-amoxiclav, cephalosporins, and macrolides [30]. In our study, the decrease in antibiotics prescribed per child probably indicates the positive results of public awareness campaigns on antibiotics use [31,32,33], as well as the introduction of a new pneumococcal conjugate vaccine, expanding serological coverage, which caused a significant decrease in the development of severe and non-severe pneumococcal infections and the associated antibiotic prescriptions for treatment. [34,35] Third, different rapid point-of-care tests with high sensitivity and specificity have been developed to distinguish between bacterial and viral aetiology for some specific conditions, and the number of users has increased by 5% yearly since 2014 in North America [36]. Still, there is no homogeneous access to and use of immunological and microbiological rapid point-of-care testing in the primary care settings among different countries [37], limiting the generalizability and the comparison of prescribing behaviour.

Similar to our previous study [38], amoxicillin prescriptions for pharyngitis represented only 30% of total antibiotic use, with the rest comprising co-amoxiclav. This inappropriate prescribing behaviour was observed previously and it does not seem to have declined. Moreover, co-amoxiclav and third-generation cephalosporins were prescribed together, and treatment switches between these classes were not rare. This may reflect an insufficient knowledge of molecule coverage [39,40,41] or, more likely, the patient’s non-adherence due to compliance. In fact, because of the oral suspension formulation, palatability directly affects children’s compliance, and it has been determined that in some cases, children prefer the taste and smell of cephalosporin formulations [42]. Our study has shown the high use of macrolides as first-line therapy for lower respiratory tract infections, in about 10% of cases of which, co-amoxiclav and/or third-generation cephalosporins were prescribed concomitantly. Lower respiratory tract infections are mainly caused by viruses, such as respiratory syncytial virus, influenza virus, and rhinoviruses, and when a bacterial aetiology or co-infection is suspected, amoxicillin alone should be the first-line therapy [43,44]. Moreover, the treatment patterns of bronchitis and bronchiolitis were similar to those of pneumonia, highlighting uncertainties around the differential diagnosis.

Most of the switches occurred on the eighth day after the initial antibiotic prescription. This indicates that treatment failure could be the cause of switching antibiotics, since if an adverse event occurs, the switch would likely be in the first days after the prescription. On the other hand, adverse events such as diarrhoea following high clavulanate dosages might appear around the fifth day after the treatment initiation, since the hypothesized changes in the microflora could take some time [12,45]. Other reasons that might explain an early switch are a poor compliance due to palatability, while the persistence of symptoms might be the reason for a late switch.

Several limitations need to be addressed in our study. First, even though the study was based on individual-level patient data, we were not able to retrieve a diagnosis in 16% of cases. However, we believe that missing diagnoses in a few patients will not change the overall prescribing patterns in our study. Second, due to the nature of the infectious disease, some children had multiple treatment episodes, which could lead to close monitoring by their FPs. Thus, a 14-day screening period from the initial episode and any subsequent similar diagnosis treatment during the period was considered as a follow-up visit for the same episode. It might be argued that in some cases (e.g., pneumonia and bone infections), the healing could be slower; however, we believe that this is an exception and not the rule. Moreover, by defining an episode, we were able to reduce the over-representativeness of complex patients. Third, since family paediatricians are the primary referrals for health-related matters as well as the gatekeepers for specialist referrals in the context of the NHS, we believe that loss to follow-up, in the case of treatment switching or prolongation, is rare. Indeed, most of the diagnoses considered consisted of uncomplicated community infections that are treated at the primary care level, where access to healthcare is free and granted by the NHS. Moreover, caregivers are discouraged from requiring further specialistic evaluations for uncomplicated community infections due to the long waiting lists and increased costs related to a specialist’s evaluation. However, we acknowledge this as a limitation. Fourth, most of the oral paediatric formulations are liquid formulations, and the dosage is based on the child’s body weight; thus, older children might require two packages to finish the antibiotic treatment course instead of one. According to our definition, if the second prescription was on a follow-up visit of the same treatment episode, it was reported as treatment prolongation. Fifth, data on allergies and vaccination status were not assessed. A change in these factors might have been the cause of the high broad-spectrum prescription rates or switches in therapy. Sixth, we were unable to determine the reasons for switching antibiotics in our current study. It is warranted to explore the reasons for switching antibiotics further.

## 4. Materials and Methods

### 4.1. Data Source

The Pedianet database was used as the source of the study. Pedianet is a national population database that contains anonymous patient-level data of more than 500,000 children since 2004, corresponding to around 4% of the annual paediatric population, who received healthcare from 161 family paediatricians (FPs) in Italy who were part of the Pedianet network.

The network links FPs distributed throughout several Italian regions designated by the Italian NHS, including Friuli-Venezia Giulia, Liguria, Lombardia, Piemonte, Veneto, Lazio, Marche, Toscana, Abruzzo, Campania, Sardegna, and Sicilia, and who use the same software (Junior Bit^®^) (Padova, Italy) in their professional practice. About 4500 family paediatricians use Junior Bit^®^ in Italy and they are all potential candidates to be part of the Pedianet network [46]. For this study, we included the data of 140 FPs who contributed to the database from 1 January 2012 to 31 December 2018; the data were extracted on 1 March 2019. Yearly numbers of patients by FPs are summarized in Figure S4 in the Supplementary Material.

According to the Italian NHS, each child is assigned to a FP who is the primary referral for health-related matters. In Italy, there is a tax-funded public healthcare system with universal access, and patients do not incur in any direct costs related to primary care visits [47]. The Pedianet database captures several types of patient-level information, including the reason for accessing healthcare, health status, demographic data, diagnosis and clinical symptoms (free text or ICD-9 CM codes), drugs (Anatomical-Therapeutical-Chemical codes), specialist appointments, diagnostic procedures, hospital or emergency room (ER) admissions, growth parameters, and clinical outcome data. Data are anonymized with a monthly update to a centralized database based in So.Se.Pe., the legal owner of Pedianet, in Padova. Informed consent is required from the children’s parents to enter the data in the database and to have Pedianet data linked to other databases such as the vaccine registry database or the hospitalization database using unique patient identifiers. [46] Data are manually validated for study-specific conditions, and the accuracy of the diagnosis data was verified [48,49].

### 4.2. Inclusion and Exclusion Criteria

All children aged 0 to 14 years enrolled in the Pedianet database during the study period were eligible for inclusion.

Children aged over 14 years or children with less than 14 days of follow-up from their first prescription date were excluded. Even though family paediatrician visits are free of charge in Italy, some families prefer private paediatricians. In these specific cases, even if the children are automatically assigned to an Italian NHS FP, they are followed by a private paediatrician outside the NHS; hence, in line with previous studies, to have a more precise denominator, children with less than two visits to the FPs were excluded [50]. In addition, children with a hospital or ER discharge less than 30 days before the date of their first antibiotic prescription for each treatment and those with visiting episodes with more than one diagnosis related to the same prescription were excluded (e.g., children with a diagnosis of pharyngitis and acute otitis media on the same visit).

### 4.3. Measures and Outcomes

The following definitions were applied:

1. Prescription: all prescriptions of the same antibiotic class were grouped if they occurred on the same visit (e.g., in case of two prescriptions for amoxicillin on the same visit, just one is counted as a prescription).
2. Antibiotic index (AI): the number of antibiotic prescriptions per person-year.
3. Treatment episode: all prescriptions occurring within 14 days of the first antibiotic prescription.
4. Switch: treatment episode with a second prescription different in class from the first [27]
5. Early switch: the first switch occurring within 1–3 days of the first prescription [27]
6. Late switch: the first switch occurring within 4–14 days of the first prescription [27]
7. Treatment prolongation: treatment episode with a second prescription.
8. Day of switching: difference in days between the date of the second prescription and the date of the first prescription in a switch.

The person-years of children included in the study were accumulated from either January 2012 or when the patients started receiving care. All patients were followed up until the last date of their care or the end of the study period in December 2018.

The primary outcomes considered were: (i) antibiotic index, (ii) antibiotic prescriptions rat, (iii) time to switch/prolongation, (iv) early switch rate, (v) late switch rate, and (vi) prolongation rate. Switch and treatment prolongation were only assessed for prescriptions associated with one diagnosis amongst patients who had an antibiotic prescribed for a reason other than prophylaxis and for children with at least 14 days of follow-up after the prescription index date.

Antibiotics were classified according to ATC codes as amoxicillin (J01CA04), co-amoxiclav (J01CR02), macrolides (J01FAx), third-generation cephalosporins (J01DCx), second-generation cephalosporins (J01DDx), J01XX (J01XXx), thiamphenicol (J01BA02), lincosamides (J01FFx), other aminoglycosides (J01Gx), and others (J01x). If two or more prescriptions with the same specific 7-digit ATC code were reported on the same visits for the same diagnosis, just one was considered for the purposes of the study.

All diagnoses linked to the same visit (max = 3) were classified according to the diagnostic categories for common outpatient infections. For determination of the years of age, the date of birth was approximated to 1 January and the year of age was calculated as the difference between 1 January and the date of birth for all the calendar years considered.

If two or more prescriptions occurred the same day, they were excluded from the analysis of Outcomes (iii), (iv), (v), and (vi) because the order of drug events could not be determined. Early and late switches were assessed for only the first switch in the treatment episode.

### 4.4. Statistical Analyses

The analysis had a mainly descriptive nature. Stratification was performed according to the diagnosis class, calendar year, and year of age (i.e., <1, 1, 2, 3, etc.). Categorical variables were expressed using numbers and percentages, and continuous variables were expressed as means and standard deviations (SD) or medians with interquartile ranges (IQR). Categorical variables were compared with a chi-squared test in a contingency table (r × c). Continuous variables were compared by a non-parametric Kruskal–Wallis rank sum test.

To estimate the annual changes in antibiotic prescriptions, the Mann–Kendall test was performed to determine whether the time series had a monotonic upward or downward trend and, if significant, the generalized linear Poisson regression was fitted. The Poisson model was chosen over linear regression because of the use of count data for antibiotic prescriptions (non-negative integers) and the high number of zero values in the dependent variables. The dependent variable was a count of antibiotic prescriptions, while calendar year, sex, age (either as a continuous either as a categorical variable), and geographical residency of the patient according to the ISTAT area were the covariates considered [51]. The log of person-years and the log of total antibiotics were included as offsets in the model estimating the antibiotic index and the antibiotic prescription rates respectively. Overdispersion was assessed to evaluate the goodness of fit of the model. The relative rate and the 95% confidence interval (95% CI) according to normal approximation were calculated.

A two-sided *p* < 0.05 was considered statistically significant. The analysis was performed using R statistical software–v. 3.6.2 (R Foundation for Statistical Computing, Vienna, Austria) [52]. Graphs were created using the packages “ggplot2” and “riverplot” in R.

### 4.5. Ethics

Due to the retrospective nature of the study and the use of anonymized data, no ethical consent was required to conduct the study. The study protocol and access to the data were approved by the Internal Scientific Committee of So.Se.Pe. Srl, the legal owner of Pedianet.

## 5. Conclusions and Future Implications

Our findings report an overall decrease in antibiotic prescriptions over the years, but pre-schoolers are still receiving more than one antibiotic yearly. Moreover, most of the prescribed antibiotic can be classified as broad-spectrum antibiotics (e.g., co-amoxiclav and III-gen. cephalosporins) and little to no variation was noted over the years. Estimating the drivers of antibiotic prescriptions is essential to define the area of intervention for antibiotic stewardship in primary care, either to reduce prescriptions for infection that have mainly a viral cause, or to improve treatment selection [7]. However, obtaining such specific estimates, even if highly informative, is time-consuming. One solution could be to use metrics developed using the WHO AWaRe antibiotic classification, namely the amoxicillin index (the percentage of total prescriptions accounted for by amoxicillin) and the Access to Watch index (the ratio of Access to Watch group use) [29]. These metrics are starting to be widely used worldwide because the joint interpretation of the three metrics is helping identify broad areas for national antibiotic stewardship and guideline development, even when information on indications is not available [53].

## Figures and Tables

**Table 1:** Antibiotic index relative rate and antibiotic prescription relative rate by class with 95% CI adjusted for calendar year, patients’ age, sex, and geographical location (Pedianet 2012–2018).

	Relative Risk											
	AI	Amoxicillin	Co-Amoxiclav	III-Gen. Cephalosporins	II-Gen. Cephalosporins	Macrolides	Lincosamides	Fluoroquinolones	J01XX	Thiamphenicol	Other Aminoglycosides	Other
**Time in year**	0.96 (0.96–0.97)	1.02 (1.01–1.02)	1.01 (1.01–1.02)	1 (1–1)	0.9 (0.89–0.9)	0.99 (0.99–0.99)	1.03 (0.99–1.07)	0.91 (0.85–0.96)	0.98 (0.96–1)	1.02 (1.01–1.03)	0.95 (0.94–0.96)	0.83 (0.8–0.86)
**Patients’ age in years**	0.87 (0.87–0.87)	0.94 (0.94–0.94)	1.01 (1.01–1.01)	1.01 (1.01–1.01)	0.93 (0.93–0.94)	1.04 (1.04–1.04)	1.2 (1.18–1.23)	1.13 (1.09–1.17)	1.17 (1.16–1.18)	0.94 (0.93–0.94)	1.14 (1.13–1.15)	1.07 (1.05–1.09)
**Male sex**	1.05 (1.04–1.06)	0.99 (0.97–1)	1.04 (1.03–1.05)	0.97 (0.96–0.99)	0.91 (0.89–0.94)	1.03 (1.02–1.04)	1.32 (1.13–1.54)	1.38 (1.08–1.76)	1.02 (0.94–1.1)	0.55 (0.52–0.57)	1 (0.94–1.05)	1.11 (0.97–1.26)
**Geographical location**												
North	ref	ref	ref	ref	ref	ref	ref	ref	ref	ref	ref	ref
Centre	1.72 (1.71–1.74)	0.35 (0.35–0.36)	1.8 (1.78–1.83)	1.37 (1.35–1.4)	1.59 (1.53–1.65)	0.92 (0.9–0.94)	0.38 (0.28–0.5)	4.4 (3.01–6.41)	0.51 (0.46–0.56)	1.5 (1.4–1.61)	0.71 (0.66–0.77)	0.84 (0.72–0.99)
South and islands	1.75 (1.74–1.76)	0.28 (0.28–0.29)	1.33 (1.31–1.34)	1.96 (1.93–1.99)	1.88 (1.83–1.94)	1.33 (1.32–1.35)	0.88 (0.75–1.03)	3.22 (2.27–4.57)	0.27 (0.25–0.3)	1.98 (1.87–2.09)	0.81 (0.76–0.86)	0.23 (0.19–0.28)

**Table 2:** Heat map of antibiotic treatment switching rate (overall, early, and late) stratified by diagnosis with the mean and the median day of switching. Only treatment switches with a prevalence rate by diagnosis higher than 5% are reported (Pedianet, 2012–2018).

Diagnosis	Treatment Switch	N of Total Switches (% of Overall Switches)	N of Total Switches (% of Overall Switches by Diagnosis)	N of Early Switches (% of Overall Early Switches by Diagnosis)	N of Late Switches (% of Overall Late Switches by Diagnosis)	Mean (SD)	Median (IQR)
**AOM**	Amoxicillin–co-amoxiclav	220 (1.3)	220 (9.6)	20 (5.1)	200 (10.2)	9.4 (3.7)	10 (6)
	Amoxicillin–III-gen. cephalosporins	215 (1.3)	215 (9.4)	35 (8.9)	180 (9.2)	8.8 (4.3)	10 (8)
	Amoxicillin–macrolides	144 (0.8)	144 (6.3)	22 (5.6)	122 (6.2)	8.7 (4.1)	9.5 (6.2)
	Co-amoxiclav–III-gen. cephalosporins	382 (2.2)	382 (16.7)	68 (17.3)	314 (16)	8.5 (4.2)	9 (7)
	Co-amoxiclav–macrolides	186 (1.1)	186 (8.1)	22 (5.6)	164 (8.3)	9.2 (4.1)	10 (7)
	III-gen. cephalosporins–co-amoxiclav	215 (1.3)	215 (9.4)	36 (9.2)	179 (9.1)	8.4 (4.1)	9 (7)
	III-gen. cephalosporins–Macrolides	150 (0.9)	150 (6.6)	17 (4.3)	133 (6.8)	8.7 (3.8)	9 (5.5)
**Bronchitis/bronchiolitis**	Amoxicillin–macrolides	299 (1.7)	299 (11.2)	44 (8.2)	255 (11.7)	7.6 (3.7)	7 (5.5)
	Co-amoxiclav–macrolides	480 (2.8)	480 (18)	80 (14.9)	400 (18.3)	7.6 (3.8)	7 (7)
	III-gen. cephalosporins–macrolides	247 (1.4)	247 (9.3)	37 (6.9)	210 (9.6)	7.9 (3.8)	8 (6)
	Macrolides–amoxicillin	148 (0.9)	148 (5.6)	39 (7.3)	109 (5)	7 (4.2)	6 (8)
	Macrolides–co-amoxiclav	427 (2.5)	427 (16)	87 (16.2)	340 (15.5)	6.9 (3.9)	7 (6)
	Macrolides–III-gen. cephalosporins	409 (2.4)	409 (15.3)	86 (16)	323 (14.8)	7.1 (3.9)	7 (6)
**Pharyngitis**	Amoxicillin–co-amoxiclav	285 (1.7)	285 (8.8)	33 (4.6)	252 (9.6)	9.4 (4)	10 (6)
	Amoxicillin–III-gen. cephalosporins	248 (1.4)	248 (7.6)	50 (6.9)	198 (7.6)	8.8 (4.5)	10 (9)
	Amoxicillin–macrolides	275 (1.6)	275 (8.5)	48 (6.6)	227 (8.7)	8.4 (4.2)	9 (8)
	Co-amoxiclav–III-gen. cephalosporins	411 (2.4)	411 (12.7)	123 (17)	288 (11)	7 (4.6)	7 (8)
	Co-amoxiclav–macrolides	406 (2.4)	406 (12.5)	72 (10)	334 (12.7)	8.3 (4.2)	8 (7)
	III-gen. cephalosporins–co-amoxiclav	249 (1.5)	249 (7.7)	51 (7.1)	198 (7.6)	8.3 (4.4)	9 (9)
	III-gen. cephalosporins–macrolides	268 (1.6)	268 (8.3)	28 (3.9)	240 (9.2)	8.7 (3.9)	9 (7)
**URTI**	Amoxicillin–co-amoxiclav	230 (1.3)	230 (5.1)	30 (4.2)	200 (5.1)	8.5 (4)	8.5 (8)
	Amoxicillin–macrolides	359 (2.1)	359 (8)	54 (7.6)	305 (7.8)	7.7 (3.7)	7 (6)
	Co-amoxiclav–III-gen. cephalosporins	338 (2)	338 (7.5)	75 (10.5)	263 (6.7)	7.9 (4.3)	8 (7.8)
	Co-amoxiclav–macrolides	566 (3.3)	566 (12.6)	85 (11.9)	481 (12.2)	7.9 (3.9)	8 (6)
	III-gen. cephalosporins–co-amoxiclav	234 (1.4)	234 (5.2)	37 (5.2)	197 (5)	8.7 (4)	9 (6)
	III-gen. cephalosporins–macrolides	275 (1.6)	275 (6.1)	30 (4.2)	245 (6.2)	8.4 (3.8)	8 (6)
	Macrolides–co-amoxiclav	664 (3.9)	664 (14.8)	87 (12.2)	577 (14.7)	8 (3.8)	7 (7)
	Macrolides–III-gen. cephalosporins	470 (2.7)	470 (10.4)	79 (11.1)	391 (10)	7.7 (4)	7 (7)
**NA**	Amoxicillin–co-amoxiclav	139 (0.8)	139 (5.4)	25 (4.8)	114 (5.4)	8.3 (4.2)	8 (7.5)
	Amoxicillin–macrolides	144 (0.8)	144 (5.6)	24 (4.6)	120 (5.7)	7.5 (3.8)	7 (7)
	Co-amoxiclav–III-gen. cephalosporins	261 (1.5)	261 (10.2)	55 (10.6)	206 (9.8)	7.8 (4.1)	8 (8)
	Co-amoxiclav–macrolides	418 (2.4)	418 (16.3)	75 (14.4)	343 (16.3)	7.9 (4.1)	7 (8)
	III-gen. cephalosporins–co-amoxiclav	189 (1.1)	189 (7.4)	39 (7.5)	150 (7.1)	8 (4.4)	8 (9)
	III-gen. cephalosporins–macrolides	163 (1)	163 (6.4)	24 (4.6)	139 (6.6)	7.8 (3.8)	7 (6.5)
	Macrolides–co-amoxiclav	244 (1.4)	244 (9.5)	45 (8.6)	199 (9.5)	7.7 (4.1)	7 (8)
	Macrolides–III-gen. cephalosporins	130 (0.8)	130 (5.1)	31 (6)	99 (4.7)	7.1 (4)	7 (7)

## Data Availability

The data used in this study cannot be made available in the manuscript, the supplemental files, or in a public repository due to Italian data protection laws. The anonymized datasets generated during and/or analysed during the current study can be provided on reasonable request from the corresponding author after written approval by the Internal Scientific Committee (info@pedianet.it).

## Supplementary Materials

The following are available online at https://www.mdpi.com/article/10.3390/antibiotics11010018/s1. Table S1. Population characteristics, antibiotic index and antibiotics prevalence rate stratified by calendar year. Pedianet 2012–2018; Table S2. Antibiotic index relative rate with 95% CI adjusted for patients age, sex and geographical location. Pedianet 2012–2018; Table S3. Antibiotics prevalence rate stratified by antibiotic class, patients year of age and calendar year. Pedianet 2012–2018; Table S4. Antibiotic prescription rate by class of antibiotic stratified according to diagnosis. (Pedianet 2012-2018); Table S5. Heatmap of the number of treatment changes (switch or prolongation) switch and prolongation with the relative prevalence on total treatment switch and prolongation. Pedianet, 2012-2018. Figure S1. Antibiotic index in different years stratified by patient years of age. Pedianet, 2012–2018; Figure S2. Prescription index prevalence rate of antibiotic classes for Pharyngitis (panel A), URTI (panel B), No diagnosis (panel C), bronchitis/bronchiolitis (panel D), SMMI (panel E), AOM (panel F), UTI (panel G), sinusitis (panel H), viral infections (panel I), STI (panel L), pneumonia (penal M) stratified by year and age class. Only diagnosis with prescription for all the strata are reported. Pedianet, 2012–2018; Figure S3. Treatment episodes with at least one switch stratified by diagnosis. Pedianet, 2012–2018; Figure S4. Annual number of patients followed by the 140 FPs participating in Pedianet from 2012 to 2018.
