# Supplementary material for: Antibiotic Prescribing Patterns in Paediatric Primary Care in Italy: Findings from 2012–2018
Source: Antibiotics (Basel). 2021 Dec 24;11(1):18. doi: 10.3390/antibiotics11010018 (PMC8773435; doi:10.3390/antibiotics11010018)

Supplementary material

Tables

- **Table S1.** Population characteristics, antibiotic index and antibiotics prevalence rate stratified by calendar year. Pedianet 2012-2018
- **Table S2.** Antibiotic index relative rate with 95% CI adjusted for patients age, sex and geographical location. Pedianet 2012-2018
- **Table S3.** Antibiotics prevalence rate stratified by antibiotic class, patients year of age and calendar year. Pedianet 2012-2018
- **Table S4.** Antibiotic prescription rate by class of antibiotic stratified according to diagnosis. (Pedianet 2012-2018)
- **Table S5.** Heatmap of the number of treatment changes (switch or prolongation) switch and prolongation with the relative prevalence on total treatment switch and prolongation. Pedianet, 2012-2018

Figures

- **Figure S1.** Antibiotic index in different years stratified by patient years of age. Pedianet, 2012-2018
- **Figure S2.** Prescription index prevalence rate of antibiotic classes for Pharyngitis (panel A), URTI (panel B), No diagnosis (panel C), bronchitis/bronchiolitis (panel D), SMMI (panel E), AOM (panel F), UTI (panel G), sinusitis (panel H), viral infections (panel I), STI (panel L), pneumonia (penal M) stratified by year and age class. Only diagnosis with prescription for all the strata are reported. Pedianet, 2012-2018
- **Figure S3.** Treatment episodes with at least one switch stratified by diagnosis. Pedianet, 2012-2018
- **Figure S4.** Annual number of patients followed by the 140 FPs participating in Pedianet from 2012 to 2018.

Table S1. Population characteristics, antibiotic index and antibiotics prevalence rate stratified by calendar year. Pedianet 2012-2018

	**2012**	**2013**	**2014**	**2015**	**2016**	**2017**	**2018**	p value
**Children 0-14 years of age, N**	103684	106609	107316	107707	107516	106387	104877	
**Sex, Male, N(%)**	53743 (51.8)	55315 (51.9)	55681 (51.9)	55926 (51.9)	55835 (51.9)	55175 (51.9)	54291 (51.8)	0.991
**Age, Years**								
Mean (SD)	6.54 (6.51, 6.56)	6.67 (6.65, 6.70)	6.83 (6.81, 6.86)	6.93 (6.91, 6.96)	6.99 (6.97, 7.02)	7.10 (7.08, 7.13)	7.28 (7.26, 7.30)	**< 0.001**
**Geographical location**								**< 0.001**
North, N(%)	54826 (52.9)	56205 (52.7)	56208 (52.4)	56154 (52.1)	56117 (52.2)	55597 (52.3)	54792 (52.2)	
Centre, N(%)	14816 (14.3)	14992 (14.1)	14960 (13.9)	14946 (13.9)	15061 (14.0)	14513 (13.6)	14132 (13.5)	
South with Islands, N(%)	34042 (32.8)	35412 (33.2)	36148 (33.7)	36607 (34.0)	36338 (33.8)	36277 (34.1)	35953 (34.3)	
**Follow-up, Person-years**								**< 0.001**
N	99276.49	102385.89	103786.27	103706.96	103560.21	102867.92	101618.68	
Mean (SD)	0.96 (0.16)	0.96 (0.16)	0.97 (0.14)	0.96 (0.16)	0.96 (0.15)	0.97 (0.14)	0.97 (0.14)	
**Antibiotic prescriptions**								**< 0.001**
N	78036	83085	76869	71539	70551	64846	61001	
Mean (SD)	0.75 (1.39)	0.78 (1.40)	0.72 (1.33)	0.66 (1.25)	0.66 (1.25)	0.61 (1.20)	0.58 (1.17)	
**Antibiotic index, (95%CI)**	0.80 (0.78, 0.81)	0.82 (0.81, 0.83)	0.75 (0.74, 0.76)	0.72 (0.71, 0.73)	0.71 (0.69, 0.72)	0.64 (0.63, 0.65)	0.62 (0.61, 0.63)	**< 0.001**
**Total antibiotics, N**	78036	83085	76869	71539	70551	64846	61001	
Co-amoxiclav, N(%)	24615 (32)	26120 (31)	25135 (33)	24062 (34)	23966 (34)	22005 (34)	24615 (32)	**< 0.001**
Amoxicillin, N(%)	17685 (23)	18094 (22)	15573 (20)	15191 (21)	16217 (23)	14692 (23)	17685 (23)	**< 0.001**
Macrolides, N(%)	15211 (19)	17309 (21)	17921 (23)	15177 (21)	14331 (20)	12781 (20)	15211 (19)	**< 0.001**
III-gen. Cephalosporins, N(%)	12273 (16)	13898 (17)	12212 (16)	11440 (16)	11302 (16)	10675 (16)	12273 (16)	**< 0.001**
II-gen. Cephalosporins, N(%)	5410 (7)	4518 (5)	3565 (5)	3403 (5)	2824 (4)	2441 (4)	5410 (7)	**< 0.001**
J01xx, N(%)	1092 (1)	1170 (1)	1104 (1)	1012 (1)	874 (1)	954 (1)	1092 (1)	**< 0.001**
Tiamphenicol, N(%)	135 (0)	302 (0)	160 (0)	96 (0)	77 (0)	80 (0)	135 (0)	**< 0.001**
Other aminoglycosides, N(%)	486 (1)	509 (1)	347 (0)	371 (1)	267 (0)	395 (1)	486 (1)	**< 0.001**
Fluoroquinolones, N(%)	108 (0)	122 (0)	68 (0)	80 (0)	92 (0)	114 (0)	108 (0)	0.136
Lincosamides, N(%)	45 (0)	53 (0)	56 (0)	34 (0)	26 (0)	38 (0)	45 (0)	0.065
Other, N(%)	976 (1)	990 (1)	728 (1)	673 (1)	575 (1)	671 (1)	976 (1)	**< 0.001**

Table S2. Antibiotic index relative rate with 95% CI adjusted for patients age, sex and geographical location. Pedianet 2012-2018

	**Relative rate**	**Lower CI**	**Higher CI**	**p.value**
Time in years	0.96	0.96	0.96	<0.001
Patients age in years				
<1	ref	ref	ref	-
1	1.85	1.82	1.89	<0.001
2	1.94	1.91	1.98	<0.001
3	1.97	1.93	2.01	<0.001
4	1.97	1.93	2.01	<0.001
5	1.84	1.80	1.87	<0.001
6	1.37	1.34	1.40	<0.001
7	0.99	0.97	1.01	0.219
8	0.72	0.71	0.74	<0.001
9	0.64	0.62	0.65	<0.001
10	0.58	0.57	0.59	<0.001
11	0.50	0.49	0.52	<0.001
12	0.43	0.42	0.44	<0.001
13	0.40	0.39	0.41	<0.001
Male sex	1.05	1.04	1.05	<0.001
Geographical location				
North	ref	ref	ref	-
Centre	1.73	1.72	1.75	<0.001
South with Islands	1.74	1.73	1.75	<0.001

Table S3. Antibiotics prevalence rate stratified by antibiotic class, patients year of age and calendar year. Pedianet 2012-2018

	2012<br>N(%)	2013<br>N(%)	2014<br>N(%)	2015<br>N(%)	2016<br>N(%)	2017<br>N(%)	2018<br>N(%)	Grand Total<br>N(%)
Co-amoxiclav	*24615 (15)*	*26120 (16)*	*25135 (15)*	*24062 (14)*	*23966 (14)*	*22005 (13)*	*20731 (12)*	*166634 (100)*
0	567 (17)	577 (17)	539 (16)	471 (14)	438 (13)	400 (12)	378 (11)	3370 (100)
1	2426 (16)	2602 (17)	2343 (15)	2099 (14)	2170 (14)	1978 (13)	1768 (11)	15386 (100)
2	2887 (15)	3060 (16)	2890 (15)	2633 (14)	2459 (13)	2538 (14)	2229 (12)	18696 (100)
3	3236 (16)	3014 (15)	3235 (16)	3030 (15)	2886 (14)	2489 (12)	2552 (12)	20442 (100)
4	3257 (15)	3580 (16)	3319 (15)	3220 (15)	3219 (15)	2888 (13)	2410 (11)	21893 (100)
5	2779 (15)	2768 (15)	2974 (16)	2662 (14)	2842 (15)	2507 (13)	2444 (13)	18976 (100)
6	2114 (14)	2314 (15)	2033 (14)	2142 (14)	2280 (15)	1988 (13)	2086 (14)	14957 (100)
7	1416 (13)	1678 (15)	1543 (14)	1578 (14)	1676 (15)	1535 (14)	1498 (14)	10924 (100)
8	1325 (14)	1460 (15)	1401 (14)	1402 (14)	1447 (15)	1349 (14)	1308 (13)	9692 (100)
9	1290 (15)	1256 (15)	1250 (15)	1216 (14)	1225 (14)	1168 (14)	1153 (13)	8558 (100)
10	1191 (16)	1321 (17)	1009 (13)	1043 (14)	1037 (14)	1034 (14)	914 (12)	7549 (100)
11	866 (14)	1066 (17)	1070 (17)	898 (14)	863 (14)	853 (13)	763 (12)	6379 (100)
12	715 (14)	768 (15)	811 (16)	882 (17)	702 (13)	723 (14)	610 (12)	5211 (100)
13	546 (12)	656 (14)	718 (16)	786 (17)	722 (16)	555 (12)	618 (13)	4601 (100)
Amoxicillin	*17685 (16)*	*18094 (16)*	*15573 (14)*	*15191 (14)*	*16217 (15)*	*14692 (13)*	*13964 (13)*	*111416 (100)*
0	727 (19)	667 (18)	600 (16)	479 (13)	482 (13)	407 (11)	387 (10)	3749 (100)
1	2690 (17)	2814 (18)	2385 (15)	2054 (13)	2040 (13)	2008 (13)	1706 (11)	15697 (100)
2	2602 (17)	2475 (16)	2384 (15)	2261 (14)	2018 (13)	1950 (12)	1965 (13)	15655 (100)
3	2435 (16)	2527 (17)	2164 (14)	2183 (14)	2214 (14)	1822 (12)	1949 (13)	15294 (100)
4	2279 (15)	2321 (16)	2160 (15)	1981 (13)	2206 (15)	2103 (14)	1690 (11)	14740 (100)
5	1714 (15)	1742 (15)	1530 (13)	1617 (14)	1894 (16)	1575 (13)	1606 (14)	11678 (100)
6	1214 (15)	1220 (15)	1050 (13)	1127 (14)	1427 (17)	1171 (14)	1118 (13)	8327 (100)
7	810 (14)	902 (15)	714 (12)	757 (13)	991 (17)	919 (15)	842 (14)	5935 (100)
8	697 (15)	763 (16)	558 (12)	621 (13)	761 (16)	700 (15)	695 (14)	4795 (100)
9	752 (18)	646 (15)	498 (12)	527 (12)	596 (14)	594 (14)	617 (15)	4230 (100)
10	649 (18)	701 (19)	428 (12)	454 (12)	516 (14)	503 (14)	451 (12)	3702 (100)
11	478 (15)	567 (18)	479 (15)	404 (13)	404 (13)	396 (13)	381 (12)	3109 (100)
12	349 (15)	430 (18)	320 (13)	402 (17)	313 (13)	295 (12)	297 (12)	2406 (100)
13	289 (14)	319 (15)	303 (14)	324 (15)	355 (17)	249 (12)	260 (12)	2099 (100)
Macrolides	*15211 (15)*	*17309 (17)*	*17921 (17)*	*15177 (14)*	*14331 (14)*	*12781 (12)*	*12011 (11)*	*104741 (100)*
0	295 (15)	370 (19)	282 (14)	285 (14)	273 (14)	248 (13)	213 (11)	1966 (100)
1	1290 (16)	1406 (17)	1368 (16)	1086 (13)	1130 (14)	1132 (14)	902 (11)	8314 (100)
2	1545 (15)	1693 (16)	1624 (16)	1515 (15)	1393 (13)	1336 (13)	1225 (12)	10331 (100)
3	1984 (16)	2049 (16)	2077 (17)	1860 (15)	1661 (13)	1515 (12)	1310 (11)	12456 (100)
4	2010 (15)	2250 (17)	2258 (17)	2068 (15)	1865 (14)	1724 (13)	1455 (11)	13630 (100)

5	1874 (16)	1835 (15)	2091 (17)	1715 (14)	1657 (14)	1442 (12)	1395 (12)	12009 (100)
6	1365 (15)	1632 (17)	1472 (16)	1333 (14)	1284 (14)	1128 (12)	1145 (12)	9359 (100)
7	896 (13)	1116 (17)	1153 (17)	913 (14)	924 (14)	860 (13)	820 (12)	6682 (100)
8	840 (14)	1040 (17)	1075 (17)	855 (14)	823 (13)	717 (12)	808 (13)	6158 (100)
9	913 (16)	902 (16)	1030 (18)	806 (14)	744 (13)	618 (11)	651 (11)	5664 (100)
10	747 (14)	965 (18)	964 (18)	742 (14)	716 (13)	610 (11)	594 (11)	5338 (100)
11	602 (13)	810 (17)	980 (21)	623 (13)	622 (13)	566 (12)	537 (11)	4740 (100)
12	466 (11)	678 (16)	823 (19)	782 (18)	556 (13)	460 (11)	502 (12)	4267 (100)
13	384 (10)	563 (15)	724 (19)	594 (16)	683 (18)	425 (11)	454 (12)	3827 (100)
III-gen. cephalosporins	*12273 (15)*	*13898 (17)*	*12212 (15)*	*11440 (14)*	*11302 (14)*	*10675 (13)*	*9995 (12)*	*81795 (100)*
0	231 (19)	242 (19)	191 (15)	179 (14)	161 (13)	133 (11)	111 (9)	1248 (100)
1	1142 (16)	1215 (17)	1110 (16)	1045 (15)	953 (13)	877 (12)	804 (11)	7146 (100)
2	1444 (15)	1667 (17)	1458 (15)	1354 (14)	1275 (13)	1240 (13)	1133 (12)	9571 (100)
3	1645 (16)	1744 (17)	1586 (15)	1476 (14)	1443 (14)	1315 (13)	1252 (12)	10461 (100)
4	1599 (15)	1910 (17)	1609 (15)	1552 (14)	1521 (14)	1483 (14)	1283 (12)	10957 (100)
5	1446 (16)	1494 (16)	1406 (15)	1254 (13)	1367 (15)	1189 (13)	1161 (12)	9317 (100)
6	1042 (14)	1194 (17)	1044 (15)	1044 (15)	994 (14)	986 (14)	895 (12)	7199 (100)
7	766 (14)	928 (17)	785 (15)	728 (14)	751 (14)	703 (13)	698 (13)	5359 (100)
8	603 (13)	765 (16)	704 (15)	624 (13)	655 (14)	655 (14)	643 (14)	4649 (100)
9	668 (16)	637 (15)	562 (14)	535 (13)	577 (14)	595 (14)	547 (13)	4121 (100)
10	656 (17)	748 (19)	509 (13)	480 (12)	500 (13)	496 (13)	485 (13)	3874 (100)
11	463 (14)	621 (19)	509 (16)	419 (13)	430 (13)	386 (12)	395 (12)	3223 (100)
12	314 (12)	432 (16)	431 (16)	415 (16)	347 (13)	365 (14)	324 (12)	2628 (100)
13	254 (12)	301 (15)	308 (15)	335 (16)	328 (16)	252 (12)	264 (13)	2042 (100)
II-gen. cephalosporins	*5410 (22)*	*4518 (19)*	*3565 (15)*	*3403 (14)*	*2824 (12)*	*2441 (10)*	*2154 (9)*	*24315 (100)*
0	106 (27)	78 (20)	48 (12)	53 (13)	31 (8)	52 (13)	29 (7)	397 (100)
1	621 (23)	491 (18)	388 (14)	395 (15)	292 (11)	275 (10)	241 (9)	2703 (100)
2	769 (22)	627 (18)	515 (15)	484 (14)	374 (11)	351 (10)	347 (10)	3467 (100)
3	955 (24)	679 (17)	581 (15)	573 (15)	463 (12)	374 (9)	321 (8)	3946 (100)
4	803 (21)	762 (20)	532 (14)	550 (15)	458 (12)	388 (10)	298 (8)	3791 (100)
5	674 (22)	521 (17)	470 (16)	411 (14)	379 (13)	301 (10)	262 (9)	3018 (100)
6	409 (20)	377 (18)	308 (15)	295 (14)	257 (12)	198 (10)	225 (11)	2069 (100)
7	309 (22)	263 (19)	191 (14)	185 (13)	175 (12)	155 (11)	135 (10)	1413 (100)
8	197 (20)	203 (21)	145 (15)	122 (13)	97 (10)	106 (11)	103 (11)	973 (100)
9	168 (22)	126 (17)	93 (12)	99 (13)	106 (14)	78 (10)	78 (10)	748 (100)
10	173 (26)	149 (23)	96 (15)	69 (10)	64 (10)	71 (11)	39 (6)	661 (100)
11	106 (20)	128 (24)	79 (15)	82 (16)	51 (10)	47 (9)	35 (7)	528 (100)
12	58 (17)	63 (19)	64 (19)	54 (16)	39 (12)	27 (8)	28 (8)	333 (100)
13	62 (23)	51 (19)	55 (21)	31 (12)	38 (14)	18 (7)	13 (5)	268 (100)

Fluroquinolones	*108 (16)*	*122 (18)*	*68 (10)*	*80 (12)*	*92 (14)*	*114 (17)*	*94 (14)*	*678 (100)*
0	3 (30)	0 (0)	0 (0)	0 (0)	4 (40)	1 (10)	2 (20)	10 (100)
1	5 (8)	13 (22)	2 (3)	9 (15)	11 (19)	4 (7)	15 (25)	59 (100)
2	15 (27)	3 (5)	5 (9)	4 (7)	12 (21)	9 (16)	8 (14)	56 (100)
3	11 (23)	10 (21)	1 (2)	2 (4)	3 (6)	18 (38)	2 (4)	47 (100)
4	6 (16)	2 (5)	11 (29)	2 (5)	3 (8)	3 (8)	11 (29)	38 (100)
5	2 (5)	6 (15)	3 (8)	7 (18)	11 (28)	6 (15)	5 (13)	40 (100)
6	7 (16)	4 (9)	7 (16)	2 (4)	7 (16)	16 (36)	2 (4)	45 (100)
7	1 (4)	6 (26)	2 (9)	8 (35)	0 (0)	2 (9)	4 (17)	23 (100)
8	9 (36)	1 (4)	3 (12)	2 (8)	1 (4)	3 (12)	6 (24)	25 (100)
9	12 (18)	33 (51)	1 (2)	5 (8)	3 (5)	5 (8)	6 (9)	65 (100)
10	10 (22)	12 (26)	3 (7)	2 (4)	7 (15)	10 (22)	2 (4)	46 (100)
11	11 (18)	8 (13)	12 (19)	13 (21)	2 (3)	11 (18)	5 (8)	62 (100)
12	8 (13)	10 (16)	4 (7)	14 (23)	5 (8)	6 (10)	14 (23)	61 (100)
13	8 (8)	14 (14)	14 (14)	10 (10)	23 (23)	20 (20)	12 (12)	101 (100)
J01XX	*1092 (15)*	*1170 (16)*	*1104 (15)*	*1012 (14)*	*874 (12)*	*954 (13)*	*1045 (14)*	*7251 (100)*
0	97 (17)	124 (21)	98 (17)	77 (13)	50 (9)	62 (11)	75 (13)	583 (100)
1	202 (16)	169 (14)	165 (13)	161 (13)	136 (11)	177 (14)	230 (19)	1240 (100)
2	114 (14)	119 (15)	101 (13)	94 (12)	94 (12)	118 (15)	149 (19)	789 (100)
3	97 (13)	123 (16)	122 (16)	106 (14)	96 (13)	106 (14)	103 (14)	753 (100)
4	116 (16)	145 (19)	115 (15)	112 (15)	78 (10)	92 (12)	86 (12)	744 (100)
5	95 (15)	104 (16)	124 (20)	87 (14)	79 (13)	75 (12)	68 (11)	632 (100)
6	73 (14)	69 (13)	87 (17)	88 (17)	63 (12)	73 (14)	72 (14)	525 (100)
7	63 (15)	64 (15)	57 (13)	66 (15)	57 (13)	57 (13)	70 (16)	434 (100)
8	57 (14)	66 (17)	61 (15)	57 (14)	59 (15)	50 (13)	45 (11)	395 (100)
9	52 (16)	48 (15)	46 (14)	43 (13)	43 (13)	42 (13)	57 (17)	331 (100)
10	53 (19)	50 (18)	37 (13)	40 (14)	34 (12)	39 (14)	32 (11)	285 (100)
11	32 (15)	41 (19)	36 (16)	32 (15)	37 (17)	23 (10)	19 (9)	220 (100)
12	19 (12)	26 (16)	30 (18)	21 (13)	22 (13)	26 (16)	19 (12)	163 (100)
13	22 (14)	22 (14)	25 (16)	28 (18)	26 (17)	14 (9)	20 (13)	157 (100)
Lincosamides	*45 (17)*	*53 (20)*	*56 (21)*	*34 (13)*	*26 (10)*	*38 (14)*	*17 (6)*	*269 (100)*
0	1 (20)	1 (20)	3 (60)	0 (0)	0 (0)	0 (0)	0 (0)	5 (100)
1	11 (85)	0 (0)	1 (8)	0 (0)	0 (0)	1 (8)	0 (0)	13 (100)
2	1 (10)	2 (20)	1 (10)	0 (0)	2 (20)	2 (20)	2 (20)	10 (100)
3	0 (0)	4 (17)	7 (29)	2 (8)	1 (4)	6 (25)	4 (17)	24 (100)
4	7 (18)	4 (11)	9 (24)	8 (21)	4 (11)	4 (11)	2 (5)	38 (100)
5	2 (8)	12 (46)	4 (15)	5 (19)	3 (12)	0 (0)	0 (0)	26 (100)
6	4 (17)	7 (30)	3 (13)	1 (4)	5 (22)	3 (13)	0 (0)	23 (100)
7	0 (0)	3 (17)	1 (6)	4 (22)	1 (6)	4 (22)	5 (28)	18 (100)

8	6 (40)	2 (13)	1 (7)	1 (7)	2 (13)	3 (20)	0 (0)	15 (100)
9	1 (3)	8 (27)	5 (17)	4 (13)	1 (3)	9 (30)	2 (7)	30 (100)
10	1 (6)	2 (13)	8 (50)	1 (6)	1 (6)	2 (13)	1 (6)	16 (100)
11	4 (27)	2 (13)	1 (7)	6 (40)	2 (13)	0 (0)	0 (0)	15 (100)
12	1 (5)	6 (30)	4 (20)	2 (10)	4 (20)	2 (10)	1 (5)	20 (100)
13	6 (38)	0 (0)	8 (50)	0 (0)	0 (0)	2 (13)	0 (0)	16 (100)
Other	*976 (19)*	*990 (19)*	*728 (14)*	*673 (13)*	*575 (11)*	*671 (13)*	*590 (11)*	*5203 (100)*
0	7 (20)	8 (23)	6 (17)	3 (9)	5 (14)	1 (3)	5 (14)	35 (100)
1	48 (24)	36 (18)	26 (13)	22 (11)	26 (13)	16 (8)	29 (14)	203 (100)
2	71 (22)	47 (14)	54 (16)	36 (11)	39 (12)	51 (15)	32 (10)	330 (100)
3	84 (18)	103 (22)	56 (12)	73 (16)	52 (11)	56 (12)	39 (8)	463 (100)
4	108 (17)	128 (20)	93 (14)	78 (12)	68 (11)	96 (15)	71 (11)	642 (100)
5	91 (16)	104 (18)	86 (15)	72 (13)	67 (12)	87 (15)	66 (12)	573 (100)
6	96 (20)	88 (18)	59 (12)	66 (14)	50 (10)	63 (13)	64 (13)	486 (100)
7	75 (19)	83 (21)	35 (9)	51 (13)	38 (10)	55 (14)	53 (14)	390 (100)
8	77 (22)	60 (17)	44 (12)	44 (12)	41 (12)	45 (13)	42 (12)	353 (100)
9	61 (19)	64 (20)	38 (12)	31 (10)	22 (7)	54 (17)	44 (14)	314 (100)
10	59 (18)	61 (19)	59 (18)	46 (14)	29 (9)	35 (11)	32 (10)	321 (100)
11	69 (20)	65 (19)	45 (13)	53 (16)	27 (8)	42 (12)	39 (11)	340 (100)
12	74 (21)	75 (21)	48 (14)	36 (10)	51 (15)	28 (8)	37 (11)	349 (100)
13	56 (14)	68 (17)	79 (20)	62 (15)	60 (15)	42 (10)	37 (9)	404 (100)
Other aminoglicosides	*135 (15)*	*302 (34)*	*160 (18)*	*96 (11)*	*77 (9)*	*80 (9)*	*50 (6)*	*900 (100)*
0	0 (0)	0 (0)	2 (100)	0 (0)	0 (0)	0 (0)	0 (0)	2 (100)
1	8 (30)	14 (52)	1 (4)	1 (4)	2 (7)	0 (0)	1 (4)	27 (100)
2	3 (5)	35 (58)	9 (15)	2 (3)	7 (12)	3 (5)	1 (2)	60 (100)
3	8 (8)	40 (40)	20 (20)	8 (8)	7 (7)	12 (12)	4 (4)	99 (100)
4	15 (10)	38 (24)	28 (18)	32 (21)	14 (9)	22 (14)	7 (4)	156 (100)
5	17 (12)	52 (36)	28 (19)	19 (13)	15 (10)	8 (5)	7 (5)	146 (100)
6	11 (10)	42 (38)	22 (20)	7 (6)	10 (9)	14 (13)	6 (5)	112 (100)
7	8 (13)	15 (25)	13 (21)	7 (11)	3 (5)	6 (10)	9 (15)	61 (100)
8	11 (22)	7 (14)	7 (14)	10 (20)	10 (20)	4 (8)	0 (0)	49 (100)
9	5 (13)	10 (26)	4 (11)	2 (5)	5 (13)	7 (18)	5 (13)	38 (100)
10	18 (42)	10 (23)	5 (12)	2 (5)	2 (5)	2 (5)	4 (9)	43 (100)
11	16 (41)	18 (46)	2 (5)	0 (0)	1 (3)	0 (0)	2 (5)	39 (100)
12	4 (13)	15 (50)	3 (10)	4 (13)	0 (0)	2 (7)	2 (7)	30 (100)
13	11 (29)	6 (16)	16 (42)	2 (5)	1 (3)	0 (0)	2 (5)	38 (100)
Tiamphenicol	*486 (18)*	*509 (19)*	*347 (13)*	*371 (14)*	*267 (10)*	*395 (14)*	*350 (13)*	*2725 (100)*
0	0 (0)	0 (0)	1 (50)	0 (0)	0 (0)	0 (0)	1 (50)	2 (100)
1	4 (19)	0 (0)	4 (19)	3 (14)	0 (0)	4 (19)	6 (29)	21 (100)

2	20 (22)	11 (12)	17 (19)	17 (19)	8 (9)	3 (3)	15 (16)	91 (100)
3	36 (15)	44 (19)	30 (13)	47 (20)	27 (12)	30 (13)	19 (8)	233 (100)
4	49 (13)	79 (21)	47 (13)	50 (14)	39 (11)	53 (14)	53 (14)	370 (100)
5	60 (16)	55 (15)	45 (12)	46 (13)	44 (12)	69 (19)	46 (13)	365 (100)
6	58 (20)	58 (20)	34 (12)	41 (14)	20 (7)	44 (15)	40 (14)	295 (100)
7	39 (18)	50 (23)	18 (8)	23 (11)	18 (8)	37 (17)	28 (13)	213 (100)
8	37 (19)	38 (20)	25 (13)	26 (13)	14 (7)	31 (16)	22 (11)	193 (100)
9	39 (22)	36 (20)	20 (11)	13 (7)	14 (8)	25 (14)	31 (17)	178 (100)
10	40 (21)	30 (15)	31 (16)	29 (15)	16 (8)	27 (14)	22 (11)	195 (100)
11	37 (18)	43 (21)	28 (14)	21 (10)	17 (8)	31 (15)	25 (12)	202 (100)
12	43 (23)	36 (19)	21 (11)	20 (11)	20 (11)	20 (11)	26 (14)	186 (100)
13	24 (13)	29 (16)	26 (14)	35 (19)	30 (17)	21 (12)	16 (9)	181 (100)
Grand Total	*78036 (15)*	*83085 (16)*	*76869 (15)*	*71539 (14)*	*70551 (14)*	*64846 (13)*	*61001 (12)*	*505927 (100)*

Table S4. Antibiotic prescription rate by class of antibiotic stratified according to diagnosis. (Pedianet 2012-2018)

	Bronchitis/ bronchiolitis	Pharyngitis	Fever (unspecified)	Bacterial infection	SMMI	URTI	LRTI	UTI	Infection (unspecified)
Co-amoxiclav, (%)	15812 (24)	36383 (35)	823 (40)	291 (28)	8351 (44)	31298 (27)	879 (43)	2985 (35)	153 (39)
Amoxicillin, (%)	10186 (16)	33082 (32)	495 (24)	119 (12)	3574 (19)	22715 (20)	427 (21)	243 (3)	114 (29)
Macrolides, (%)	28760 (44)	9130 (9)	271 (13)	400 (39)	2784 (15)	35273 (31)	416 (20)	135 (2)	76 (19)
III-gen. cephalosporines, (%)	8973 (14)	18680 (18)	350 (17)	140 (14)	2506 (13)	16497 (14)	233 (11)	2293 (27)	25 (6)
II-gen. cephalosporines, (%)	1194 (2)	5298 (5)	64 (3)	36 (4)	1056 (6)	5583 (5)	74 (4)	348 (4)	13 (3)
J01xx, (%)	165 (0)	914 (1)	15 (1)	4 (0)	184 (1)	1713 (1)	3 (0)	2215 (26)	3 (1)
Thiamphenicol (%)	49 (0)	179 (0)	7 (0)	1 (0)	87 (0)	733 (1)	2 (0)	8 (0)	5 (1)
Lincosamides, (%)	5 (0)	19 (0)	1 (0)	0 (0)	8 (0)	61 (0)	0 (0)	1 (0)	0 (0)
Other aminoglycosides, (%)	17 (0)	26 (0)	0 (0)	2 (0)	17 (0)	295 (0)	1 (0)	13 (0)	0 (0)
Fluoroquinolones, (%)	31 (0)	32 (0)	1 (0)	3 (0)	44 (0)	59 (0)	0 (0)	87 (1)	1 (0)
Other antibiotics, (%)	87 (0)	318 (0)	12 (1)	27 (3)	310 (2)	1089 (1)	5 (0)	288 (3)	6 (2)

	STI	AI	BI	NA	AOM	Pneumonia	Prophylaxis	Sinusitis	Viral infection
Co-amoxiclav, (%)	1947 (51)	60 (39)	35 (49)	33584 (39)	22933 (36)	1944 (28)	620 (40)	3016 (31)	4209 (35)
Amoxicillin, (%)	526 (14)	17 (11)	9 (13)	13362 (16)	19448 (30)	856 (13)	202 (13)	2113 (22)	3004 (25)
Macrolides, (%)	667 (17)	4 (3)	13 (18)	17065 (20)	2583 (4)	2939 (43)	161 (11)	1138 (12)	2371 (20)
III-gen. cephalosporines, (%)	454 (12)	28 (18)	2 (3)	13223 (15)	13462 (21)	956 (14)	293 (19)	1801 (18)	1310 (11)
II-gen. cephalosporines, (%)	145 (4)	6 (4)	1 (1)	4394 (5)	4853 (8)	119 (2)	66 (4)	313 (3)	559 (5)
J01xx, (%)	25 (1)	0 (0)	0 (0)	1584 (2)	258 (0)	3 (0)	16 (1)	26 (0)	88 (1)
Thiamphenicol (%)	23 (1)	0 (0)	0 (0)	595 (1)	273 (0)	5 (0)	8 (1)	623 (6)	100 (1)
Lincosamides, (%)	0 (0)	0 (0)	1 (1)	105 (0)	11 (0)	1 (0)	25 (2)	18 (0)	11 (0)
Other aminoglycosides, (%)	6 (0)	0 (0)	0 (0)	335 (0)	81 (0)	2 (0)	9 (1)	42 (0)	46 (0)
Fluoroquinolones, (%)	3 (0)	3 (2)	1 (1)	239 (0)	39 (0)	10 (0)	56 (4)	61 (1)	7 (0)
Other antibiotics, (%)	48 (1)	34 (22)	10 (14)	1544 (2)	324 (1)	11 (0)	77 (5)	646 (7)	335 (3)

Table S5. Heatmap of the number of treatment changes (switch or prolongation) switch and prolongation with the relative prevalence on total treatment switch and prolongation. Pedianet, 2012-2018

	ANTIBIOTIC 2									
ANTIBIOTIC 1	Amoxicillin	Co-amoxiclav	Fluoroquinolones	II-gen. cephalosporins	III-gen. cephalosporins	J01XX	Macrolides	Other	Other aminoglicosides	**Grand Total**
**TREATMENT SWITCH AND PROLONGATION**										
Amoxicillin, N(%)	3107 (8.2)	1052 (2.8)	5 (0)	300 (0.8)	841 (2.2)	35 (0.1)	1321 (3.5)	13 (0)	8 (0)	**6682 (17.7)**
Co-amoxiclav, N(%)	522 (1.4)	8132 (21.5)	25 (0.1)	442 (1.2)	1796 (4.8)	116 (0.3)	2406 (6.4)	45 (0.1)	17 (0)	**13501 (35.7)**
Fluoroquinolones, N(%)	1 (0)	15 (0)	59 (0.2)	1 (0)	9 (0)	1 (0)	4 (0)	(0)	(0)	**90 (0.2)**
II-gen. cephalosporins, N(%)	99 (0.3)	366 (1)	2 (0)	986 (2.6)	269 (0.7)	16 (0)	338 (0.9)	9 (0)	2 (0)	**2087 (5.5)**
III-gen. cephalosporins, N(%)	245 (0.6)	1080 (2.9)	11 (0)	229 (0.6)	3890 (10.3)	42 (0.1)	1271 (3.4)	28 (0.1)	9 (0)	**6805 (18)**
J01XX, N(%)	92 (0.2)	286 (0.8)	4 (0)	40 (0.1)	124 (0.3)	81 (0.2)	149 (0.4)	14 (0)	(0)	**790 (2.1)**
Macrolides, N(%)	420 (1.1)	1679 (4.4)	9 (0)	262 (0.7)	1253 (3.3)	46 (0.1)	3900 (10.3)	22 (0.1)	6 (0)	**7597 (20.1)**
Other, N(%)	14 (0)	43 (0.1)	2 (0)	7 (0)	29 (0.1)	5 (0)	27 (0.1)	55 (0.1)	(0)	**182 (0.5)**
Other aminoglicosides, N(%)	9 (0)	10 (0)	1 (0)	2 (0)	5 (0)	(0)	1 (0)	(0)	20 (0.1)	**48 (0.1)**
**Grand Total, N(%)**	**4509 (11.9)**	**12663 (33.5)**	**118 (0.3)**	**2269 (6)**	**8216 (21.7)**	**342 (0.9)**	**9417 (24.9)**	**186 (0.5)**	**62 (0.2)**	**37782 (100)**
**ONLY TREATMENT SWITCH**										
Amoxicillin, N(%)	(0)	1049 (5.99)	5 (0.03)	300 (1.71)	840 (4.79)	35 (0.2)	1320 (7.53)	13 (0.07)	8 (0.05)	**3570 (20.37)**
Co-amoxiclav, N(%)	520 (2.97)	(0)	25 (0.14)	440 (2.51)	1792 (10.23)	115 (0.66)	2402 (13.71)	45 (0.26)	17 (0.1)	**5356 (30.57)**
Fluoroquinolones, N(%)	1 (0.01)	15 (0.09)	(0)	1 (0.01)	9 (0.05)	1 (0.01)	4 (0.02)	(0)	(0)	**31 (0.18)**
II-gen. cephalosporins, N(%)	98 (0.56)	366 (2.09)	2 (0.01)	(0)	269 (1.54)	16 (0.09)	338 (1.93)	9 (0.05)	2 (0.01)	**1100 (6.28)**
III-gen. cephalosporins, N(%)	244 (1.39)	1078 (6.15)	11 (0.06)	228 (1.3)	(0)	42 (0.24)	1270 (7.25)	28 (0.16)	9 (0.05)	**2910 (16.61)**
J01XX, N(%)	92 (0.53)	285 (1.63)	4 (0.02)	40 (0.23)	124 (0.71)	(0)	149 (0.85)	14 (0.08)	(0)	**708 (4.04)**
Macrolides, N(%)	420 (2.4)	1676 (9.57)	9 (0.05)	261 (1.49)	1252 (7.15)	46 (0.26)	(0)	22 (0.13)	6 (0.03)	**3692 (21.07)**
Other, N(%)	14 (0.08)	42 (0.24)	2 (0.01)	7 (0.04)	29 (0.17)	5 (0.03)	27 (0.15)	(0)	(0)	**126 (0.72)**
Other aminoglicosides, N(%)	9 (0.05)	10 (0.06)	1 (0.01)	2 (0.01)	5 (0.03)	(0)	1 (0.01)	(0)	0 (0)	**28 (0.17)**
**Grand Total, N(%)**	**1398 (7.98)**	**4521 (25.8)**	**59 (0.34)**	**1279 (7.3)**	**4320 (24.65)**	**260 (1.48)**	**5511 (31.45)**	**131 (0.75)**	**43 (0.25)**	**17522 (100)**

Figure S1. Antibiotic index in different years stratified by patient years of age. Pedianet, 2012-2018

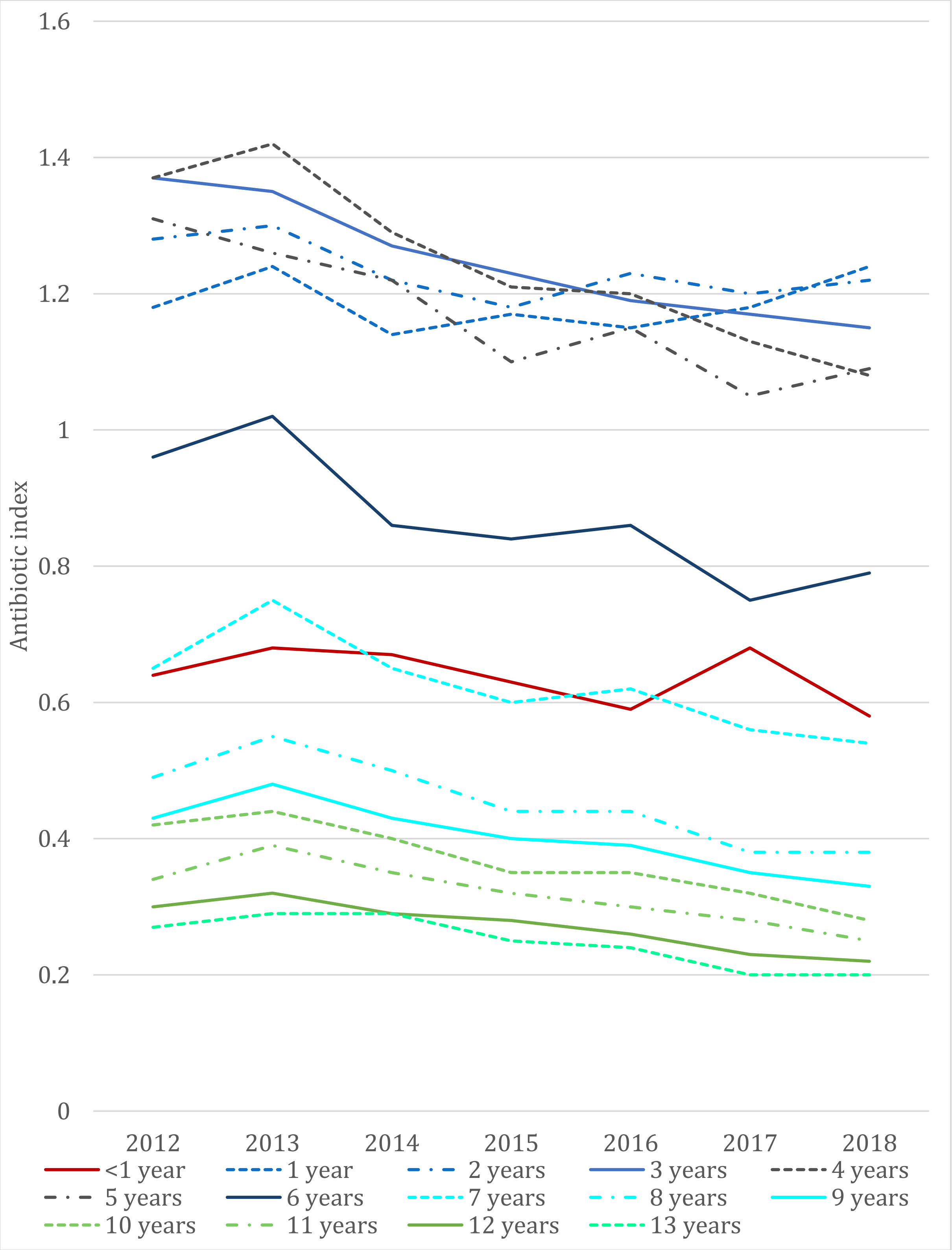

Figure S2. Figure 2s.a Prescription index prevalence rate of antibiotic classes for Pharyngitis (panel A), URTI (panel B), No diagnosis (panel C), AOM (panel D), bronchitis/bronchiolitis (panel E), SMMI (panel F), sinusitis (panel G), UTI (panel H), viral infections (panel I), STI (panel L), pneumonia (penal M) stratified by year and age class. Only diagnosis with prescription for all the strata are reported. Pedianet, 2012-2018

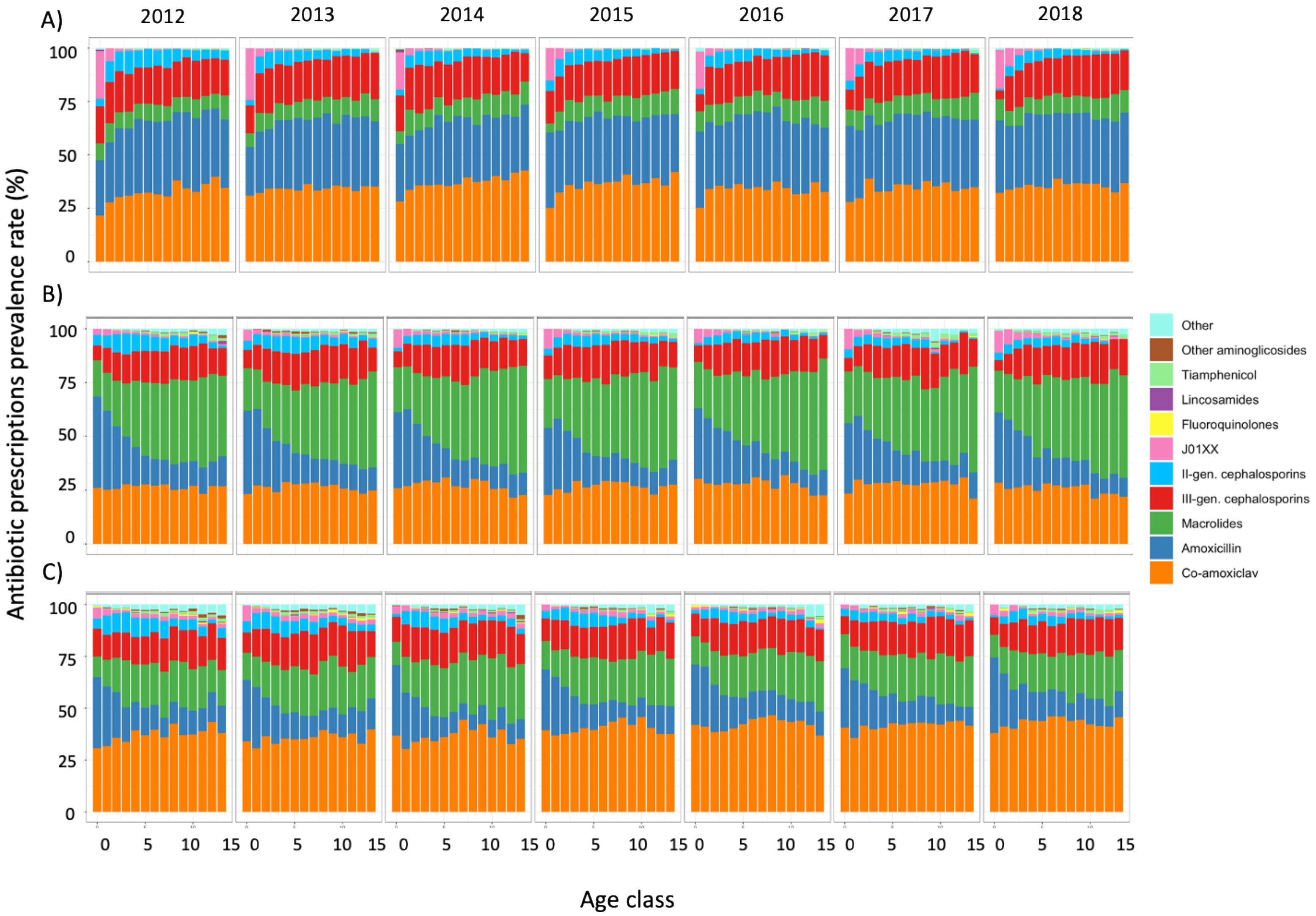

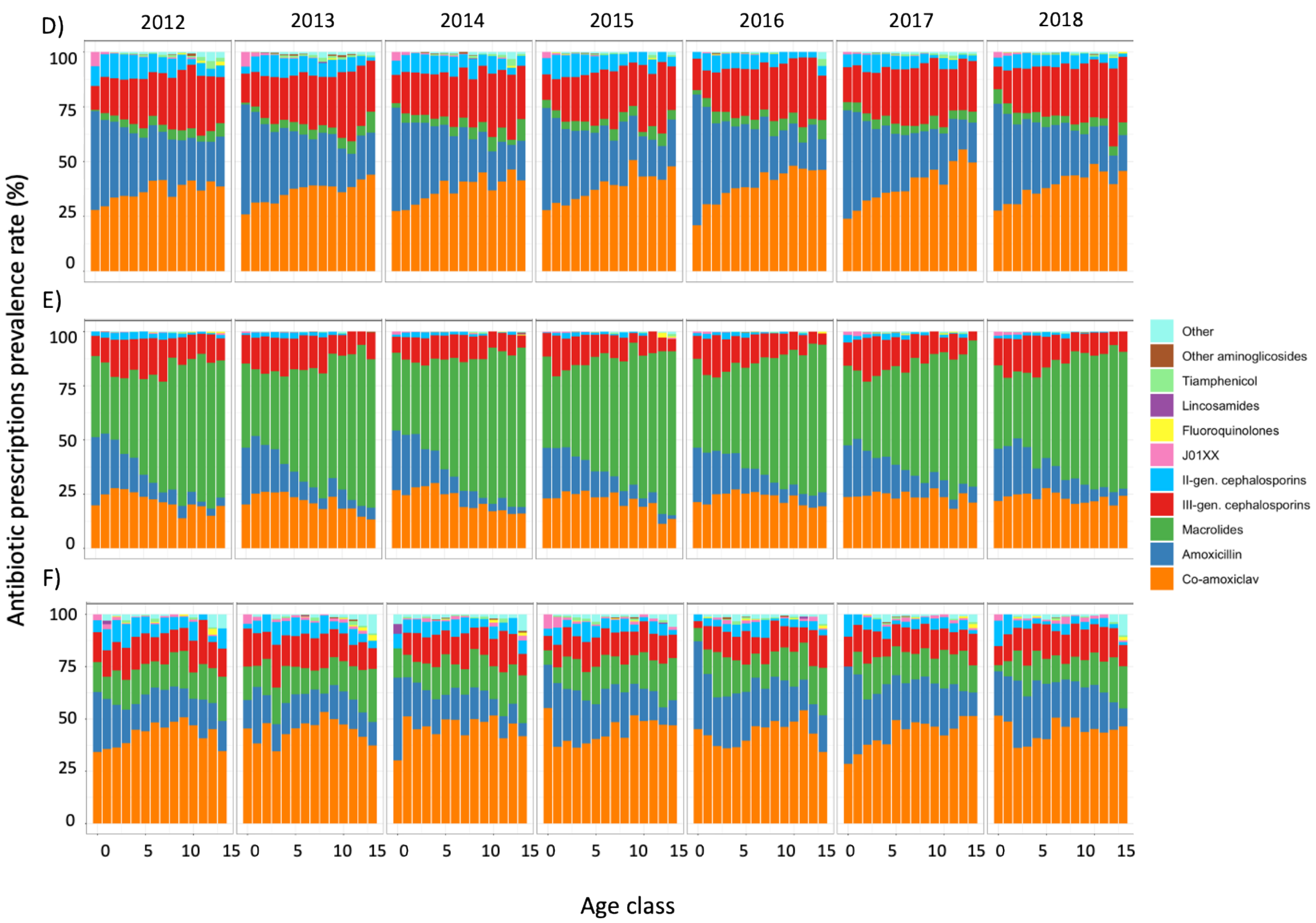
D)
E)
F)
2012
2013
2014
2015
2016
2017
2018
Antibiotic prescriptions prevalence rate (%)
100
75
50
25
0
0
5
10
15
Age class
Other
Other aminoglicosides
Tiamphenicol
Lincosamides
Fluoroquinolones
J01XX
II-gen. cephalosporins
III-gen. cephalosporins
Macrolides
Amoxicillin
Co-amoxiclav

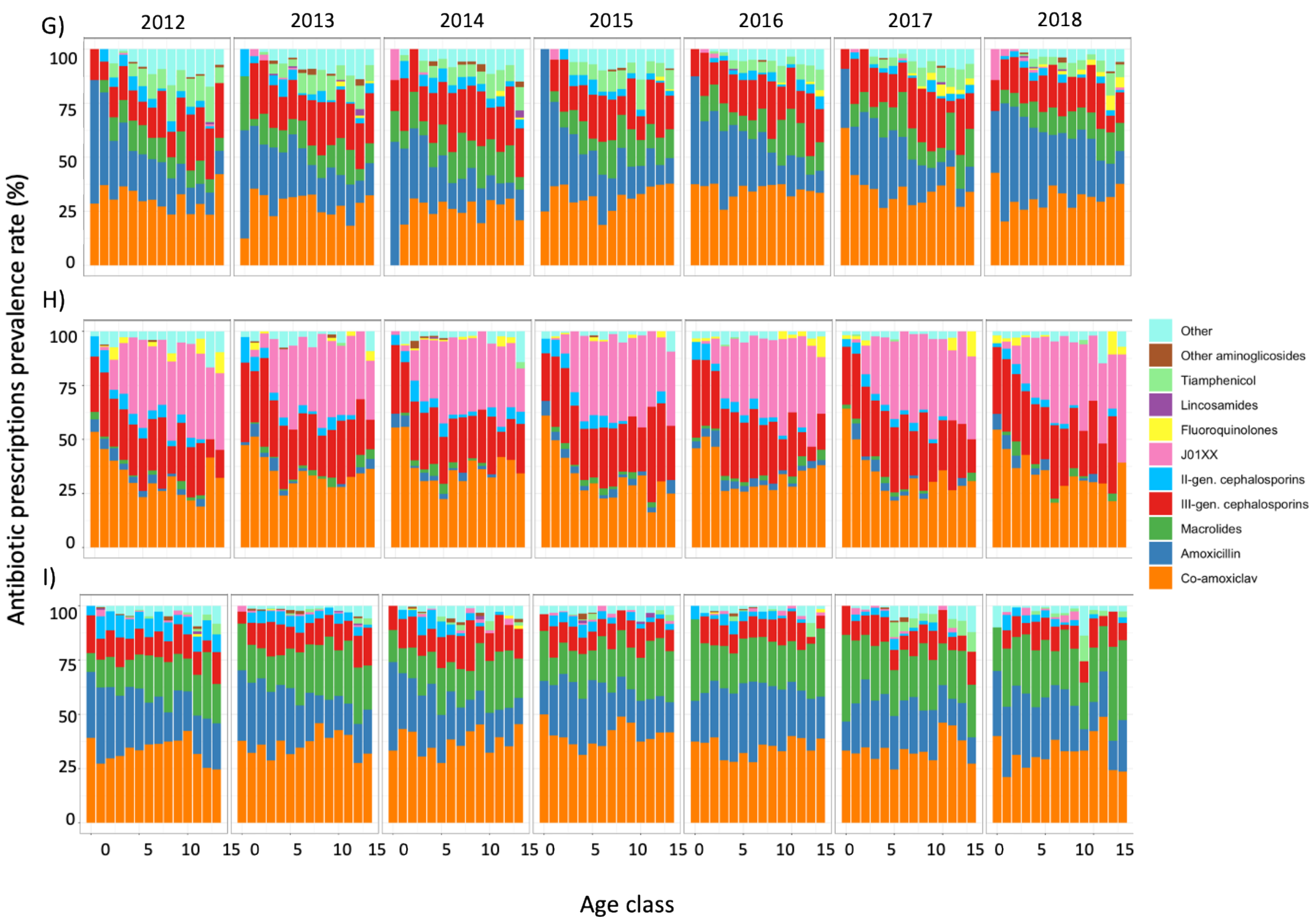

G)
H)
I)
2012
2013
2014
2015
2016
2017
2018
Antibiotic prescriptions prevalence rate (%)
100
75
50
25
0
0
5
10
15
Age class
Other
Other aminoglicosides
Tiamphenicol
Lincosamides
Fluoroquinolones
J01XX
II-gen. cephalosporins
III-gen. cephalosporins
Macrolides
Amoxicillin
Co-amoxiclav

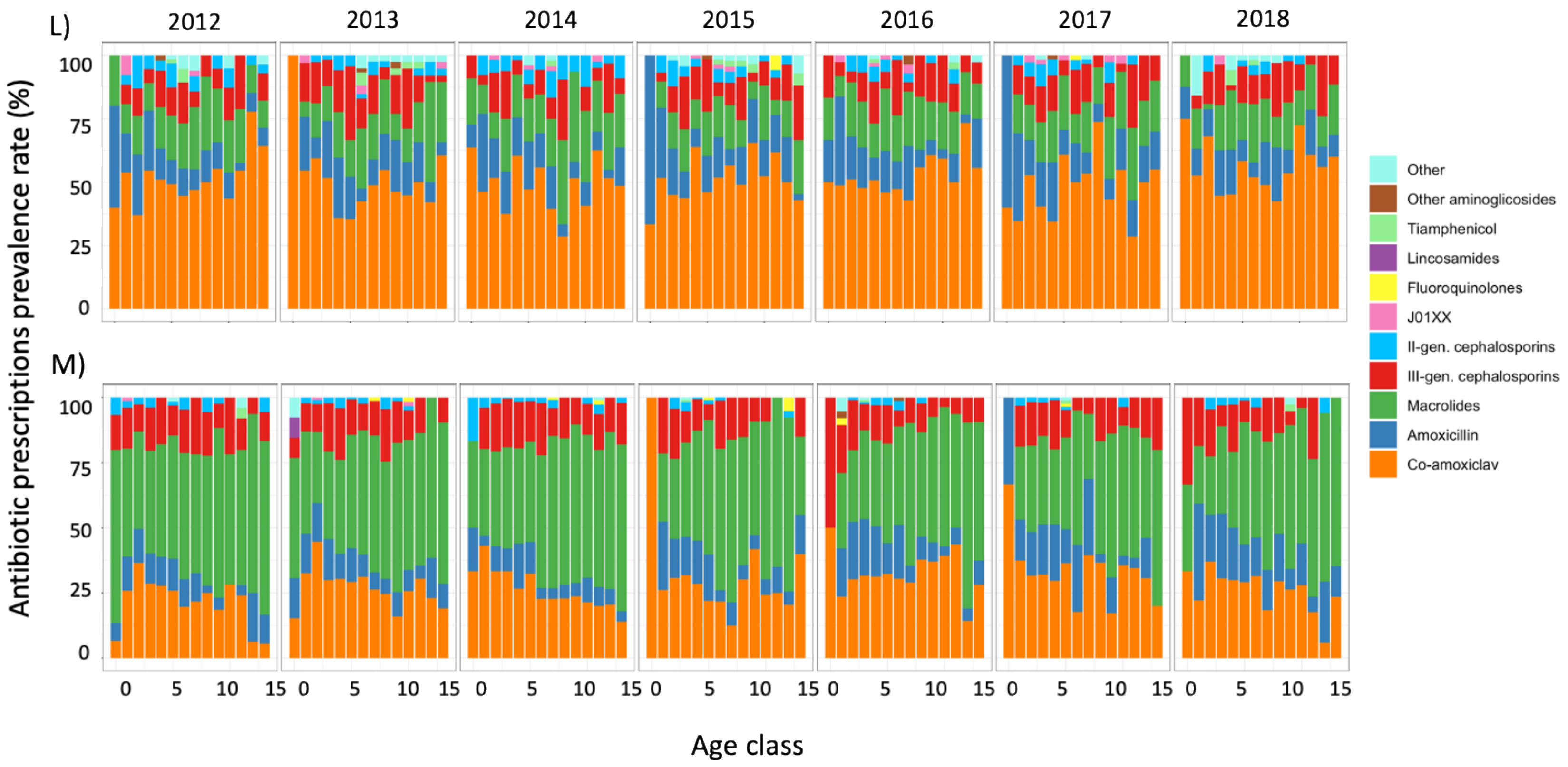
L)
M)
2012
2013
2014
2015
2016
2017
2018
Antibiotic prescriptions prevalence rate (%)
100
75
50
25
0
0
5
10
15
Age class
Other
Other aminoglicosides
Tiamphenicol
Lincosamides
Fluoroquinolones
J01XX
II-gen. cephalosporins
III-gen. cephalosporins
Macrolides
Amoxicillin
Co-amoxiclav

Figure S3. Treatment episodes with at least one switch stratified by diagnosis.

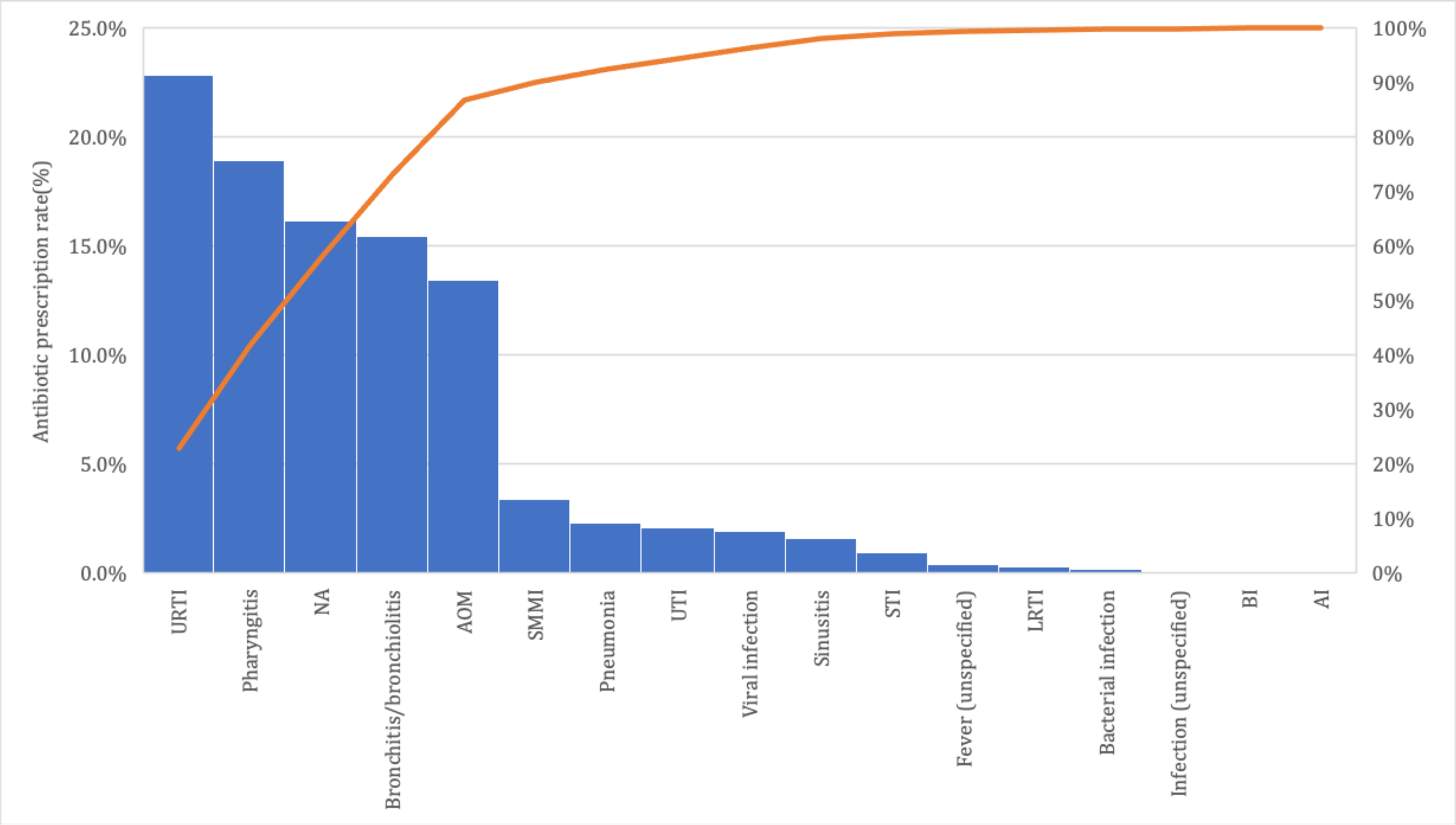

Figure S4. Annual number of patients followed by the 140 FPs participating in Pedianet from 2012 to 2018.

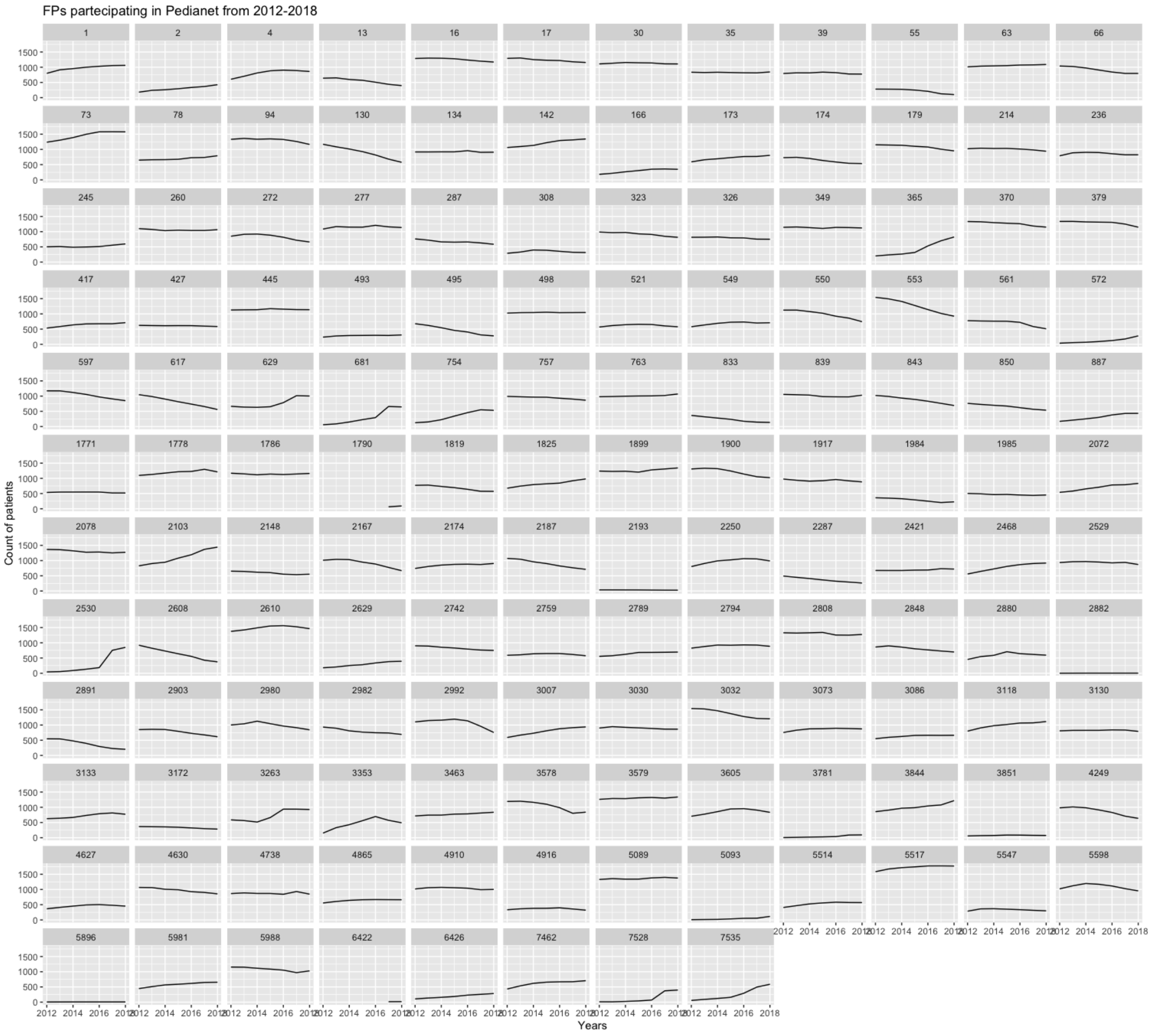

Supplement: Supplementary file 1 [file antibiotics-11-00018-s001.zip › antibiotics-1448417-supplementary.pdf]
